# Membrane curvature elastic stress triggers recruitment of PML-II onto the inner nuclear membrane

**DOI:** 10.1091/mbc.E25-09-0443

**Published:** 2025-12-10

**Authors:** Michael McPhee, Jayme Salsman, Allison A. Newman, Nikol Voutsina, Andrew H. Crosby, Graham Dellaire, Neale D. Ridgway

**Affiliations:** ^a^Department of Biochemistry and Molecular Biology, Dalhousie University, Halifax, Nova Scotia Canada B3H4R2; ^b^Department of Pathology, Dalhousie University, Halifax, Nova Scotia Canada B3H4R2; ^c^Department of Clinical and Biomedical Sciences (Medical School), Faculty of Health and Life Sciences, University of Exeter, Exeter, United Kingdom EX2 5DW; ^d^Department of Pediatrics, Dalhousie University, Halifax, Nova Scotia Canada B3H4R2; University of Alberta

## Abstract

Promyelocytic leukemia (PML) protein isoform II is a component of PML nuclear bodies (PML NBs) that also forms patches on nuclear lipid droplets (nLDs) and the inner nuclear membrane (INM). Here we tested whether different metabolic treatments that induce membrane curvature elastic stress (CES) in the INM, detected by recruitment of CTP:phosphocholine cytidylyltransferase α (CCTα) and a nuclear diacylglycerol (DAG) biosensor, are a precondition for PML-II membrane association. We found that treatment of U2OS cells with unsaturated 18-carbon fatty acids and DAG acyltransferase inhibitors caused the rapid formation of PML patches on the INM that coincided with DAG enrichment and the recruitment and stabilization of CCTα, all of which were reversed upon removal of the CES stimulus. PML patches were depleted of canonical PML NB-associated proteins, occurred at sites of lamin depletion, were specific for the PML-II isoform, and occurred in cells regardless of their capacity to assemble nLDs. Induction of INM curvature stress by knockout of the terminal enzymes of the CDP-choline pathway or lipid activators of CCTα also promoted PML patches as well as stabilization of CCTα on the INM. We conclude that CES in the INM promotes the reversible assembly of PML-II-dependent membrane-associated patches.

## Abstract (executive summary)

- Lipid metabolic stress conditions promote the association of the PML-II isoform with the inner nuclear membrane. The membrane composition that triggers PML-II association is unknown.
- Metabolic conditions that promote membrane curvature elastic stress, reported by CCTα and a DAG sensor, correlate with reversible formation of PML patches on the inner nuclear membrane at sites of nuclear lamina depletion.
- Curvature stress at the inner nuclear membrane triggers the activation of CCTα, phosphatidylcholine synthesis and PML patches to restore membrane integrity.

## INTRODUCTION

The nuclear envelope (NE) consists of an outer and inner membrane separated by a luminal space but joined at the perimeter of nuclear pore complexes (NPC). The outer nuclear membrane (ONM) is contiguous with and shares features of the endoplasmic reticulum (ER). On the other hand, the inner nuclear membrane (INM) has a unique proteome that provides structural support and genomic organization (Swift *et al.*, 2013; Ungricht and Kutay, 2017). The INM lipidome also imparts a unique membrane interface with the nuclear compartment (Bahmanyar and Schlieker, 2020; Romanauska and Kohler, 2021; Hirano *et al.*, 2024; Zhang *et al.*, 2024; Lagace and Ridgway, 2005b) that is influenced by restricted lipid exchange with the ONM at NPCs (Barger *et al.*, 2022), and compartmentalized glycerolipid and phospholipid biosynthesis (Peterfy *et al.*, 2005; Santos-Rosa *et al.*, 2005; O'Hara *et al.*, 2006; Ohsaki *et al.*, 2016; Romanauska and Kohler, 2018). For example, the INM has an evolutionarily conserved capacity for the synthesis and storage of triacylglycerol (TAG) in nuclear lipid droplets (nLDs; reviewed in Fujimoto, 2022; McPhee *et al.*, 2022). TAG synthesis in the INM of hepatoma and U2OS cells involves glycerol-3-phosphate acyltransferases 3 and 4, 1-acyl-glycerol-phosphate acyltransferases, the phosphatidic acid (PA) phosphatase Lipin1 and DAG acyltransferases (DGAT) 1 and 2 (Lee *et al.*, 2020; Soltysik *et al.*, 2021). The terminal enzymes for phosphatidylcholine (PC) and phosphatidylethanolamine (PE) synthesis have been detected at the INM in yeast (Smoyer *et al.*, 2016) but not in mammalian cells.

Lipid synthesis and/or transport at the INM results in fluctuations in PA (Soltysik *et al.*, 2021), DAG (Lee *et al.*, 2020), phosphatidylserine (Tsuji *et al.*, 2019; Niu *et al.*, 2024) and phospholipid unsaturation (Romanauska and Kohler, 2023) that affect a variety of nuclear functions (reviewed in Bahmanyar and Schlieker, 2020; Niu and Balla, 2025). Enrichment of the INM in phospholipids containing unsaturated fatty acids and amphiphiles with small polar headgroups, such as DAG, PA, and PE, can cause localized membrane curvature and induce curvature elastic stress (CES) in planar membranes (Bigay and Antonny, 2012). The perimeter of NPCs where the INM and ONM join is a region of high membrane curvature promoted by phospholipid unsaturation (Romanauska and Kohler, 2023). Recognition of these membrane junctions by nuclear pore scaffold proteins containing curvature-sensing motifs facilitates NPC assembly (Doucet *et al.*, 2010; Nordeen *et al.*, 2020). The repair of membrane herniations during defective NPC assembly involves recruitment of the ESCRT protein Chmp7 (Charged Multivesicular Body Protein 7) that binds PA (Thaller *et al.*, 2021). In planar regions of the INM, CES caused by non-bilayer lipids results in gaps between phospholipid headgroups that are recognized by amphipathic helices, leading to recruitment and regulation of protein function (Bigay and Antonny, 2012; Barger *et al.*, 2022). For example, the proteolytic degradation of SUN2, a component of the linker of nucleoskeleton and cytoskeleton (LINC) complex, is inhibited by insertion of its amphipathic helix into the DAG-enriched INM (Lee *et al.*, 2023). The nucleoplasmic enzyme CTP:phosphocholine cytidylyltransferase (CCT)α also senses CES in the INM to control the rate of synthesis of PC by the CDP-choline pathway. CCTα has a C-terminal amphipathic helix that inserts into membranes with CES packing defects caused by a reduced ratio of PC to DAG and PE (Arnold and Cornell, 1996; Cornell, 1998; Haider *et al.*, 2018). The resulting increase in CCTα activity drives PC synthesis and DAG consumption to restore planar membrane structure. Increased INM localization and expression of CCTα as a consequence of reduced PC synthesis in choline/ethanolamine phosphotransferase 1 (CEPT1) knockout U2OS cells was reversed by incubation with PC liposomes, evidence that CES at the INM is regulated by PC content (Dorighello *et al.*, 2023).

Altered lipid metabolism also causes promyelocytic leukemia nuclear bodies (PML NB) to reorganize onto the surface of the INM and nLDs. The PML gene encodes isoforms I-VII that oligomerize via RING-B-box-coiled-coil (RBCC) motifs and small-ubiquitin modifier (SUMO)-SUMO interaction motifs to form the phase-separated shell of PML NBs (Sahin *et al.*, 2014; Hoischen *et al.*, 2018). Client proteins, including transcription factors, chromatin modifiers, and SUMOylation machinery, are recruited to PML NBs to regulate DNA damage responses (Dellaire and Bazett-Jones, 2004), antiviral responses (Everett and Chelbi-Alix, 2007), apoptosis (Pearson and Pelicci, 2001), and senescence (Bischof *et al.*, 2002). Jul-Larsen *et al.* (2010) reported that ectopically expressed PML isoform II (PML-II) localized to the INM, and that 3% of U2OS cells had PML patches on the INM. PML-II also associates with the surface monolayer of nLDs in oleate-treated U2OS, Huh7 and Caco2 cells to form a unique lipid-associated PML structure (LAPS; Ohsaki *et al.*, 2016; Soltysik *et al.*, 2019; Lee *et al.*, 2020; McPhee *et al.*, 2024b). PML-II-dependent association with nLDs results in structural reorganization as indicated by the loss of the canonical PML NB client proteins SUMO, DAXX and SP100 (Lee *et al.*, 2020).

The type of lipid composition that promotes the reorganization of PML NBs into these unusual membrane bilayer- and monolayer-associated structures is unknown. A clue to the lipid compositional trigger comes from the observation that PML association with the positively curved surface of nLDs occurs in conjunction with CCTα, a known CES sensor, and enrichment in DAG (Ohsaki *et al.*, 2016; Lee *et al.*, 2020), a lipid that promotes CES. Treatment of Huh7 or Caco2 cells with oleate and DGAT inhibitors also caused PML patches on the INM concurrent with the enrichment of CCTα and DAG (McPhee *et al.*, 2024b). As well, PML-II associates with the Type-I nucleoplasmic reticulum (NR), a tubular invagination of the INM (McPhee et al., 2024a), during nLD assembly in Huh7 cells (Soltysik *et al.*, 2019). Here, we used conditions promoting CES at the INM, such as DGAT inhibition, PC deficiency, and non-bilayer membrane modifiers, to demonstrate a temporal relationship between PML-II patches, DAG enrichment, and the activation and protection of CCTα from proteolytic degradation by the proteosome. These results indicate a coordinated response to CES in the INM involving the formation of PML-II-dependent membrane patches and activation of CCTα and PC synthesis to restore membrane structure.

## RESULTS

### Inhibition of DGAT1 and 2 promotes CCTα and PML association with the INM

**FIGURE 1:**
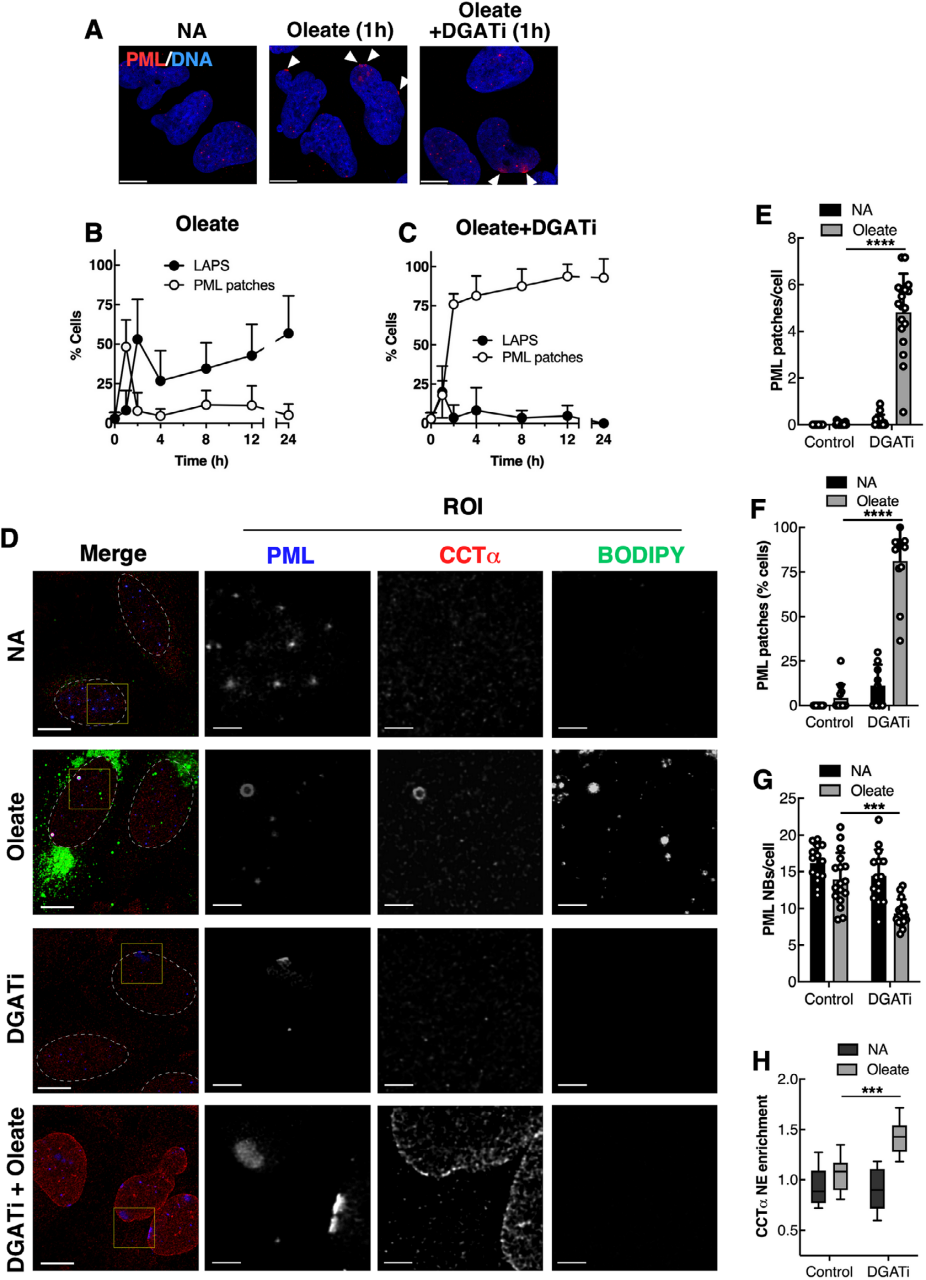
DGATi and oleate treatment of U2OS cells promotes PML and CCTα association with the INM. (A) U2OS cells treated for 1 h with no addition (NA), oleate (500 µM), DGATi (10 µM) or oleate plus DGATi (10 µM) were immunostained for PML. DNA was visualized with Hoechst 33342 (bar, 10 µm). (B and C) cells were treated with oleate (B) or oleate plus DGATi (C) for up to 24 h, immunostained as described in panel A, and the percentage of cells positive for LAPS and PML patches was quantified. Each time point represents 30–50 cells from a representative experiment. (D) U2OS cells treated as described in panel A for 16 h were immunostained with antibodies against PML and CCTα. LDs were visualized with BODIPY 493/503 (bar, 10 µm). The nucleus is outlined, and regions of interest (ROI) are to the right (bar, 2 µm). (E, F, G and H) quantification of PML patches per cell (E), percentage of cells with PML patches (F), PML NBs per cell (G) and NE enrichment of CCTα (H). Results in E–H are the mean and SD from 9 to 12 fields of cells (6–10 cells/field) per treatment from three independent experiments. Statistical significance determined by two-way ANOVA with multiple comparisons. ****p < 0.0001, ***p< 0.001.

We initially examined how lipid stress linked to CES affects PML recruitment to the INM by treating U2OS cells with oleate in the presence or absence of DGAT inhibitors. Treatment of U2OS cells with oleate resulted in transient PML patch formation on the INM at 1 h, followed by a rapid appearance of LAPS from 2 to 24 h (Figure 1, A and B). The transient appearance of PML patches on the INM was not associated with recruitment of CCTα or the DAG sensor, both of which accumulate on the surface of nLDs after oleate treatment (Ohsaki *et al.*, 2016; Lee *et al.*, 2020). To preserve the lipid environment that elicited PML patches on the INM, DGAT inhibitors were included with oleate to prevent TAG and nLD biogenesis. Based on [^3^H]oleate incorporation, DGAT1 (iDGAT1) or DGAT2 (iDGAT2) inhibition reduced TAG synthesis by 60 or 0%, respectively, but a combination of both inhibitors (DGATi) reduced TAG synthesis by >95% (Supplemental Figure 1A). Inhibition of TAG synthesis by DGATi prevented LAPS formation and caused PML patches to appear in 20% of cells at 1 h and >75% after 2 h (Figure 1C). Immunofluorescence confocal microscopy was used to quantify the INM localization of PML relative to CCTα in U2OS cells treated with DGATi and/or oleate for 16 h (Figure 1D). DGATi did not increase PML patches relative to control and oleate-treated cells, but >75% of DGATi and oleate-treated cells had 4–6 PML patches per cell (Figure 1, E and F), which was accompanied by a significant reduction in PML NBs from an average of 16 to 11 per cell (Figure 1G). DGATi and oleate treatment also caused the uniform NE enrichment of CCTα (Figure 1, D and H), indicating that CES in the INM is a potential factor in PML patch formation (Attard *et al.*, 2000; Chong *et al.*, 2014; Haider *et al.*, 2018).

**FIGURE 2:**
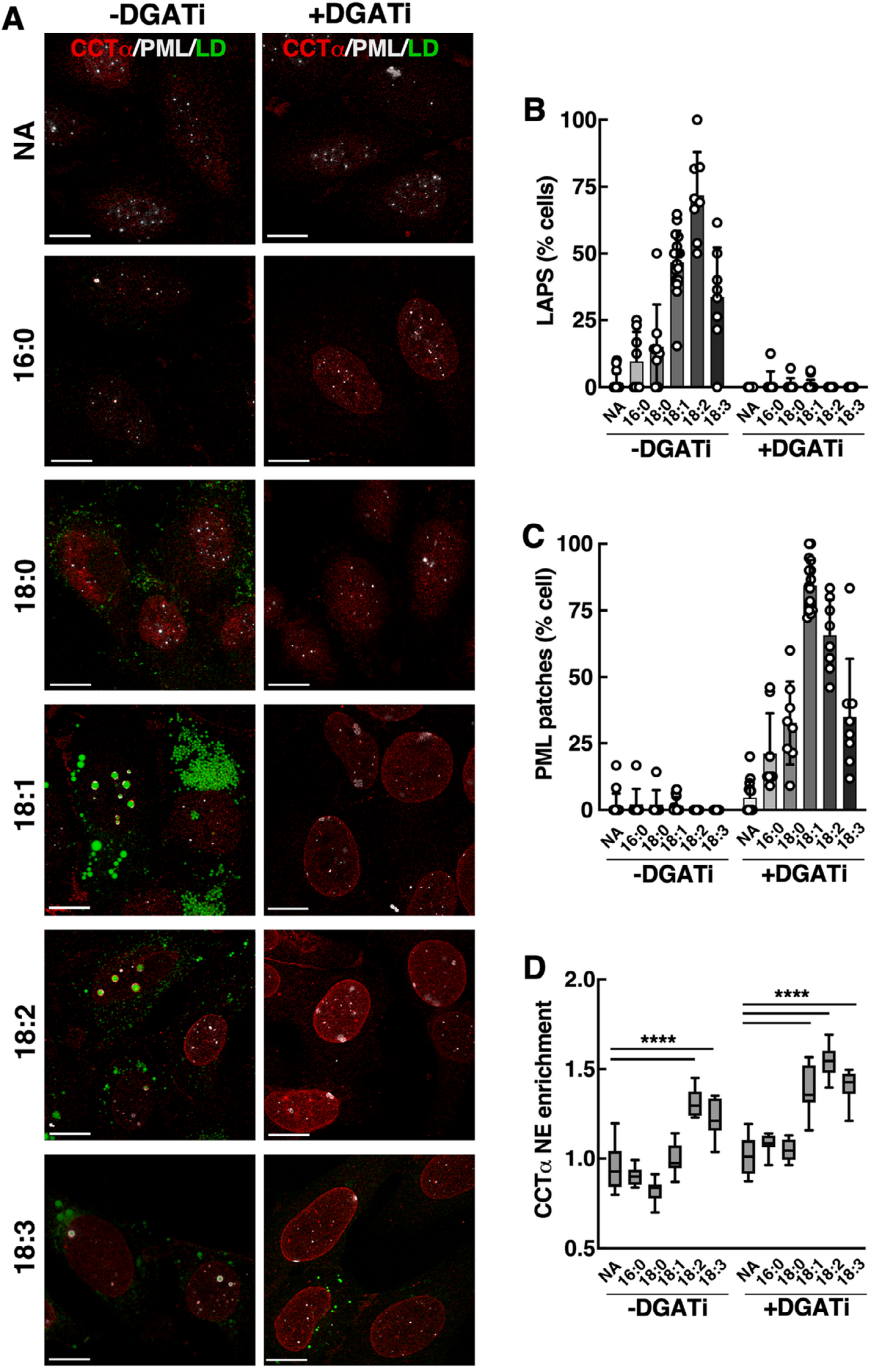
Unsaturated fatty acids and DGATi promote PML patch formation and CCTα enrichment of the INM. (A) U2OS cells treated with no addition or 500 µM palmitate (16:0), stearate (18:0), oleate (18:1), linoleate (18:2), or α-linolenate (18:3) in the absence or presence of DGATi for 24 h were immunostained for PML and CCTα, and LDs visualized with BODIPY 493/503 (bar, 10 µm). The percentage of cells with LAPS (B) and PML patches (C), and NE enrichment of CCTα (D) were quantified. Results are the mean and SD 8–16 fields of cells (*n* = 1057) from two experiments. Statistical significance determined using two-way ANOVA with multiple comparisons. ****p< 0.0001.

The effect of fatty acid unsaturation on PML recruitment to the INM was tested in U2OS cells treated with palmitate (16:0), stearate (18:0), oleate (18:1), linoleate (18:2), or linolenate (18:3) in the presence or absence of DGATi. Cells treated with oleate, linoleate or linolenate contained more LAPS versus cells treated with the saturated fatty acids palmitate or stearate (Figure 2, A and B). However, linolenate was less effective compared with oleate and linoleate. Linoleate and linolenate also promoted CCTα translocation to the INM, indicating activation by unsaturated fatty acids or CES due to their incorporation into phospholipids (Figure 2, A and D). The frequency of PML patches in cells treated with fatty acids and DGATi followed the trend observed for LAPS formation in oleate-treated cells (Figure 2C). However, it was notable that stearate was as effective as linolenate in promoting PML patches (Figure 2C). The NE enrichment of CCTα was significantly increased by all three unsaturated fatty acids in the presence of DGATi. We also assessed the effects of unsaturated fatty acids on CCTα and PML in Huh7 cells (Supplemental Figure 2). Similar to results with U2OS cells (Figure 2), oleate and linoleate caused the most significant increase in PML patch formation in Huh7 cells treated with DGATi, which correlated with NE enrichment of CCTα. U2OS and Huh7 cells treated with linoleate or linolenate alone resulted in NE enrichment of CCTα but not PML, indicating that the PML patches occur specifically in response to membrane stress induced by sustained inhibition of TAG synthesis.

**FIGURE 3:**
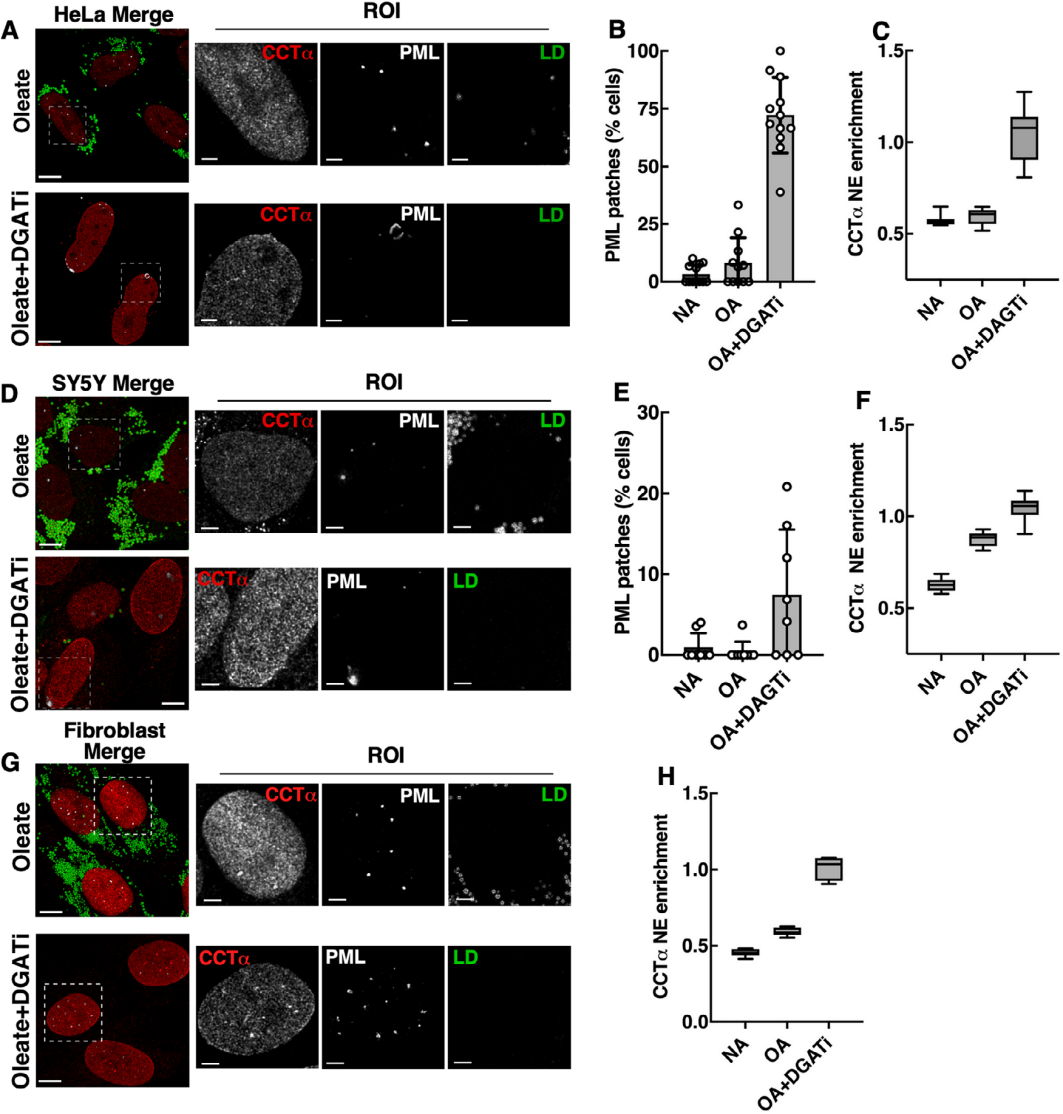
PML patch formation in cells that lack nLD or LAPS. Cells received no addition (NA), oleate (500 µM) or oleate plus DGATi for 24 h followed by immunostaining for PML and CCTα, and LD visualization with BODIPY 493/503 (bar, 10 µm). PML patches and CCTα nuclear enrichment was quantified from images. (A) Merged images of CCTα, PML and LDs in HeLa cells (bar, 10 µm) with regions of interest (ROI) highlighted (bar, 2 µm). (B) Percent HeLa cell PML patches is the mean and SD of 5 to 7 fields of cells (*n* = 320) from a representative experiment. (C) HeLa cell NE enrichment of CCTα is the mean and SD of 11 to 13 fields of cells (*n* = 619) from two independent experiments. (D) Merged images of CCTα, PML and LDs in SH-SY5Y cells with regions of interest (ROI) highlighted (bar, 10 µm). (E) Percentage of SH-SY5Y cells with PML patches (F) and NE enrichment of CCTα. Results are the mean and SD of 7 to 8 fields of cells (*n* = 633) from a representative experiment. (G) Merged images of CCTα, PML and LDs in human fibroblasts cells with regions of interest (ROI) highlighted (bar, 10 µm). (H) NE enrichment of CCTα is the mean and SD of 6 fields of cells (*n* = 249) from a representative experiment.

Human primary skin fibroblasts, HeLa and SH-SY5Y cells were chosen to test whether PML patches are induced in cells that do not assemble nLDs or LAPS. Approximately 75% of oleate and DGATi-treated HeLa cells had PML patches (Figure 3, A and B) and significant NE enrichment of CCTα (Figure 3, A and C). PML patches were evident in only ∼12% of SH-SY5Y cells treated with DGATi and oleate, but CCTα NE enrichment was significantly increased (Figure 3, D–F). In fibroblasts, DGATi and oleate treatment promoted CCTα enrichment on the NE and nucleoplasmic structures that were positive for PML, but PML patches on the INM were not evident (Figure 3, G and H). Thus, inhibition of TAG synthesis is consistently associated with NE enrichment of CCTα, while formation of PML patches on the INM was variable and not restricted to cells that assemble nLDs and LAPS.

### PML patches are depleted of canonical PML nuclear body proteins and nuclear lamina

**FIGURE 4:**
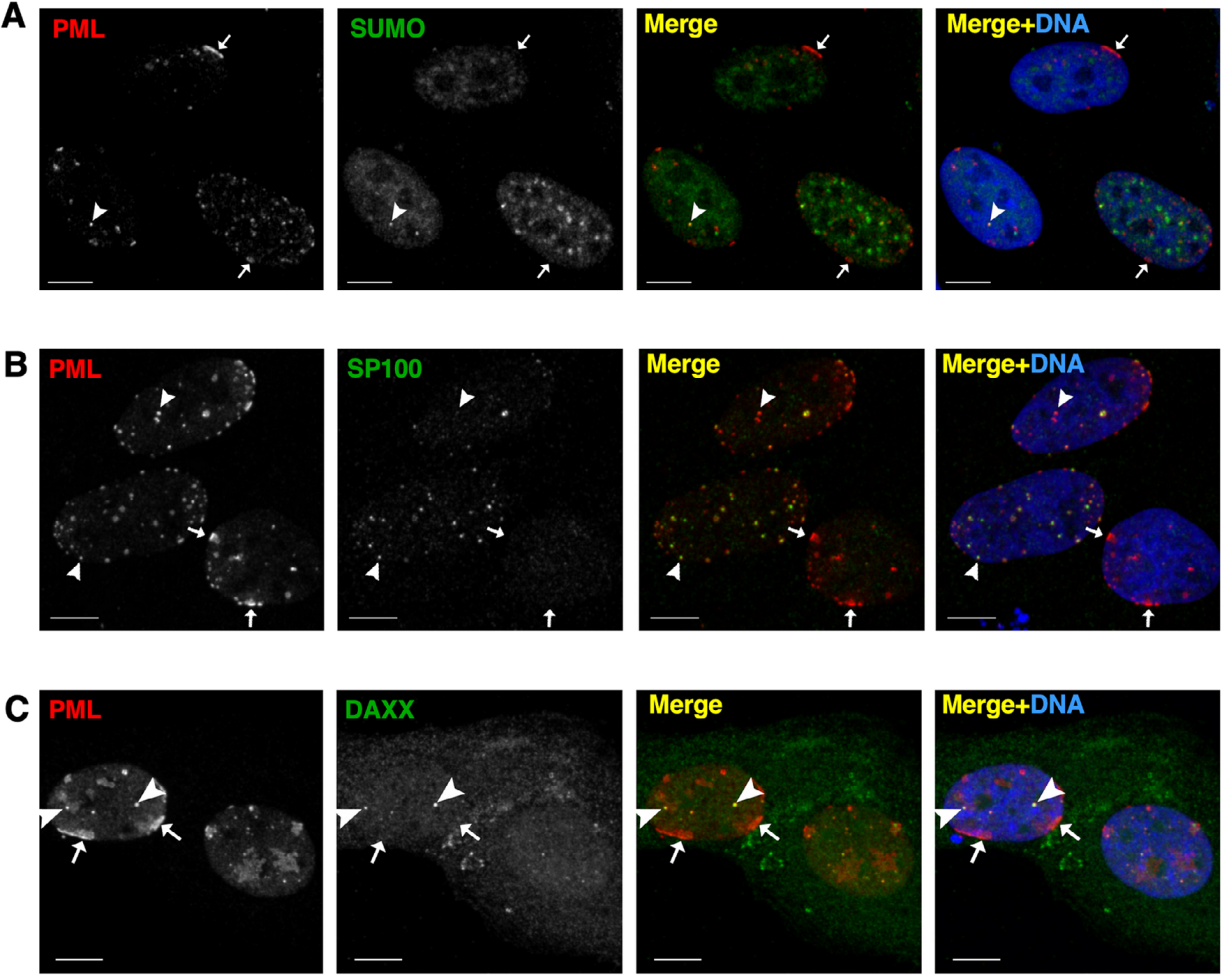
PML patches are depleted of canonical PML NB-associated proteins. U2OS cells were treated with DGATi and oleate for 16 h and immunostained with antibodies against SUMO (A), SP100 (B) or DAXX (C). The nucleus was visualized with DAPI in the merged image. Small arrows point to PML patches and large arrows point to PML NBs (bar, 10 µm).

PML NBs are dynamic structures that can reorganize under specific cellular conditions into alternate structures, each with a different repertoire of associated client proteins, such as PML clastasomes (Janer *et al.*, 2006) and alternate lengthening of telomere-associated PML NBs (Chung *et al.*, 2012). Similarly, LAPS are deficient in the canonical PML NB client proteins DAXX, SUMO and SP100 (Lee *et al.*, 2020). Confocal imaging of oleate and DGATi-treated U2OS cells indicated that PML patches were also deficient in SUMO, SP100, and DAXX (indicated by small arrows in Figure 4, A–C) compared with PML NBs in the nucleoplasm (indicated by large arrows in Figure 4, A–C). To determine whether deSUMOylation promoted PML patch formation, U2OS cells were treated with the SUMO activating enzyme inhibitor ML-792 and the presence of PML structures, including patches and nuclear bodies, was compared in U2OS cells treated with oleate or oleate plus DGATi (Supplemental Figure 3). Inhibition of SUMOylation reduced all PML-positive nuclear structures, with the remainder being large PML inclusions and threads (Supplemental Figure 3, A and B). ML-792 caused a significant reduction in SUMO-positive PML structures that was greater than that observed with oleate and DGATi treatment (Supplemental Figure 3, A and C). However, ML-792 caused only a 10% increase in PML patches compared with 75% in cells treated with DGATi and oleate (Supplemental Figure 2D), indicating that deSUMOylating per se does not promote the nucleation of PML patches.

**FIGURE 5:**
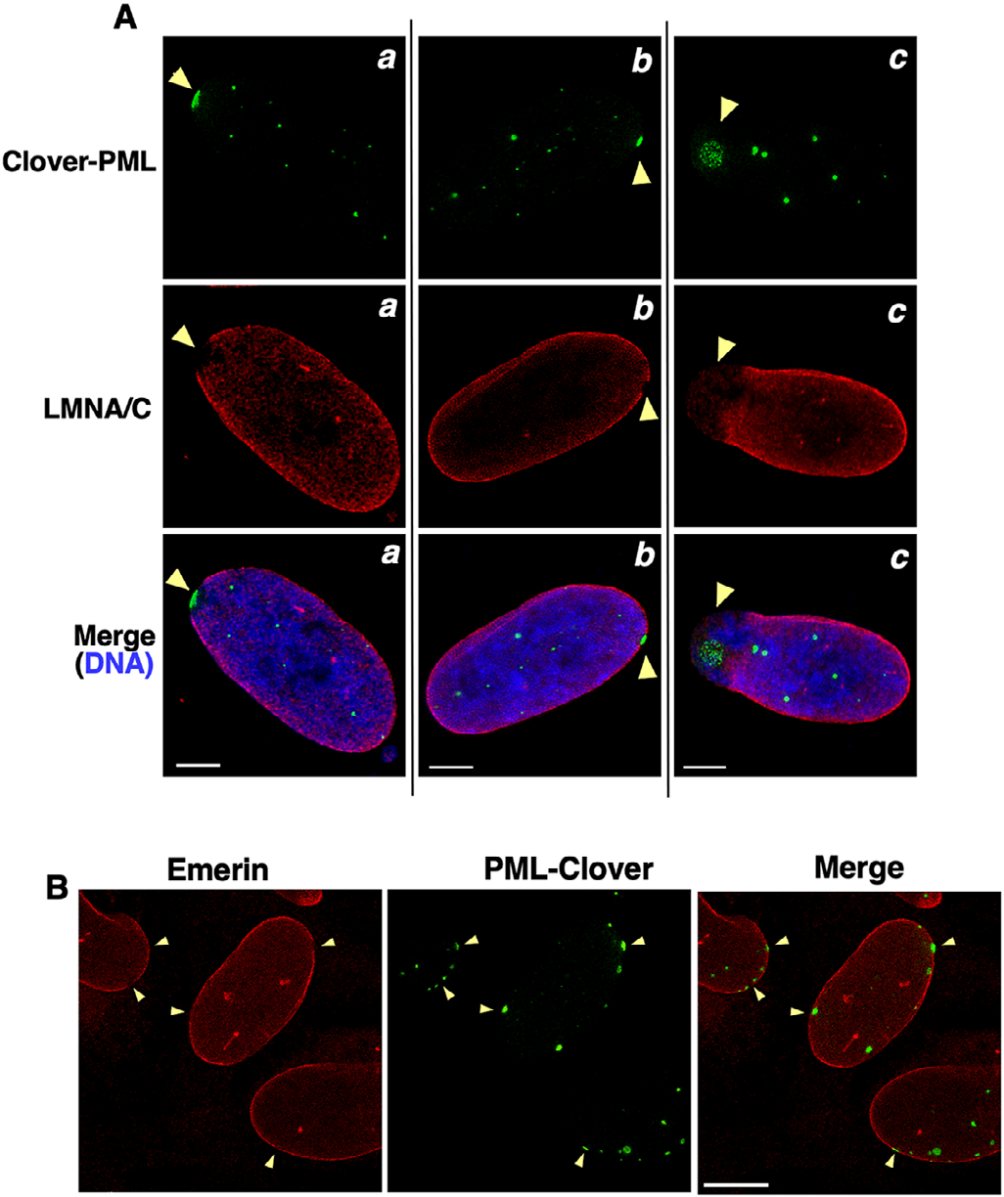
PML patches are zones of nuclear lamina depletion. (A) Clover-PML U2OS cells treated with DGATi and oleate for treated for 24 h were immunostained for LMNA/C and DNA was visualized with Hoechst 33342 (bar, 5 µm). (B) Clover-PML U2OS cells treated as described above were immunostained for emerin (bar, 10 µm). Arrows indicate PML patches at regions of LMNA/C and emerin depletion.

Ectopically expressed PML-II causes patches on the INM that are relatively devoid of nuclear lamina (Jul-Larsen *et al.*, 2010; Ohsaki *et al.*, 2016). U2OS cells with a CRISPR knock-in of the Clover green fluorescent protein at exon 1 of the PML gene (U2OS Clover-PML) were treated with oleate and DGATi to determine whether endogenous PML patches were also depleted of LMNA/C (Figure 5A) and emerin (Figure 5B). First, small Clover-PML patches coincided with regions of the NE that were relatively devoid of LMNA/C (Figure 5A, panels a and b) and emerin (Figure 5B). Secondly, a small percentage of cells (<10%) contained large Clover-PML patches associated with regions of the NE that were devoid of both LMNA/C and chromatin staining (Figure 5A, panel c).

### Localization of PML isoform II to the INM is enhanced in DGATi and oleate-treated cells

**FIGURE 6:**
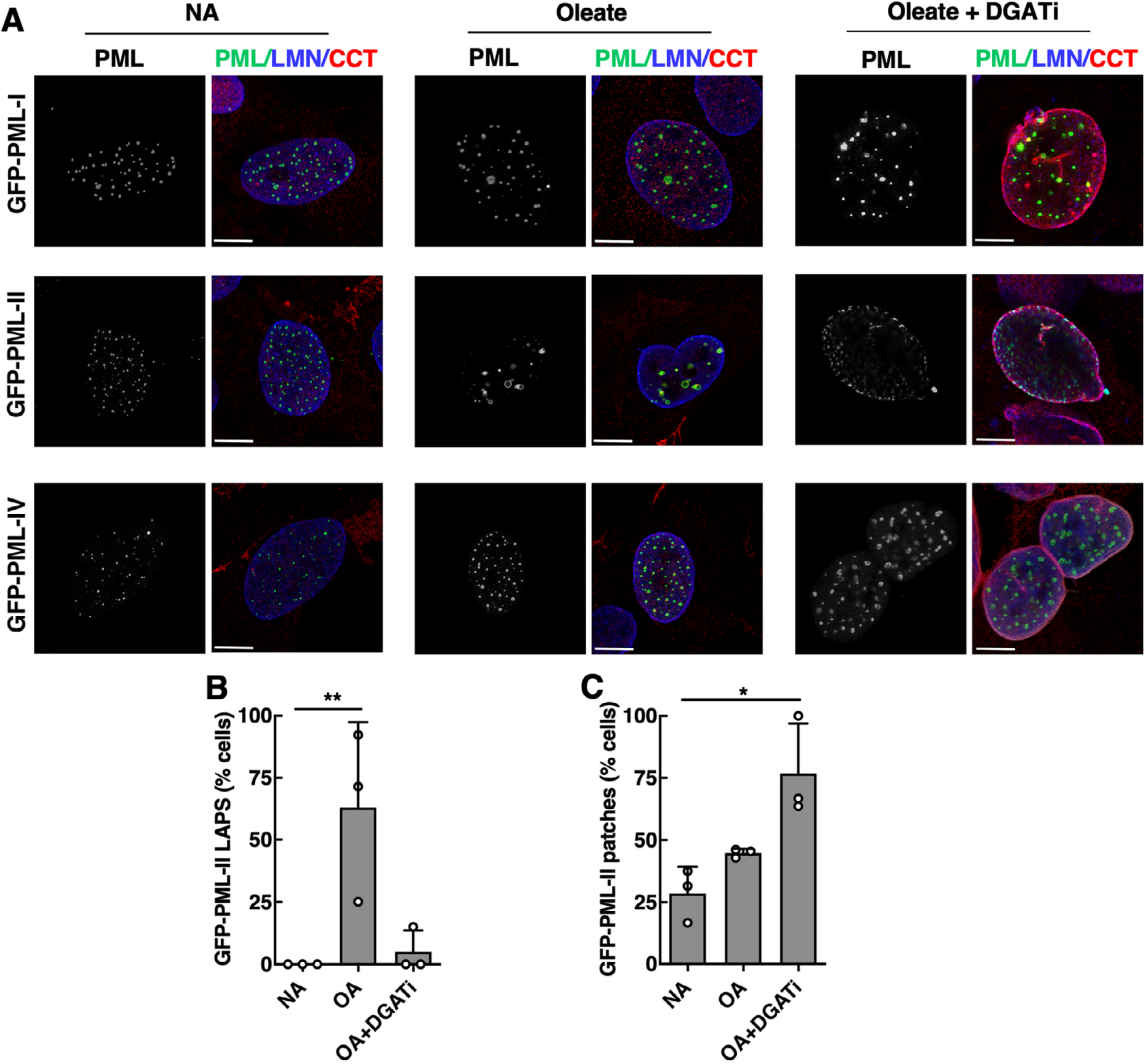
PML-II forms patches on the INM during inhibition of TAG synthesis. (A) U2OS PML KO cells transiently expressing GFP-tagged PML-I, PML-II, or PML-IV were treated with no addition (NA), oleate, or oleate and DGATi for 16 h and immunostained with antibodies against LMNA/C and CCTα (bar, 10 µm). The percentage of GFP-expressing cells (*n* = 163) with (B) LAPS (identified as ring structures with associated GFP-PML and CCTα) and (C) PML patches were quantified. Results are the mean and SD of three independent experiments. Statistical significance assessed using Student's test. *p< 0.05, **p< 0.01.

Since PML-II specifically promotes nLD biogenesis and targets the INM (Ohsaki *et al.*, 2016; Lee *et al.*, 2020), we tested whether PML-II is also required for patch formation in U2OS cells treated with DGATi and oleate. GFP-PML-I, -PML-II, and -PML-IV were transiently expressed in PML-KO U2OS cells treated with no addition, oleate or DGATi plus oleate and their localization, together with endogenous CCTα, was determined by confocal microscopy. GFP-PML-II but not GFP-PML-I or PML-IV localized to LAPS or the INM in oleate- or oleate plus DGATi-treated cells, respectively (Figure 6A). Approximately 60% of oleate-treated cells contained GFP-PML-II-positive LAPS, which was almost completely prevented by inclusion of DGATi (Figure 6B). GFP-PML-II formed patches at the INM in 25% and 40% of control or oleate-treated cells, respectively. DGATi and oleate treatment significantly increased the frequency of GFP-PML-II patches to 75% and caused the robust localization of CCTα to the ΝΕ (Figure 6C). Thus, partitioning of overexpressed PML-II into the INM is significantly enhanced by oleate and DGATi.

### Association of CCTα with the INM prevents proteasomal degradation

**FIGURE 7:**
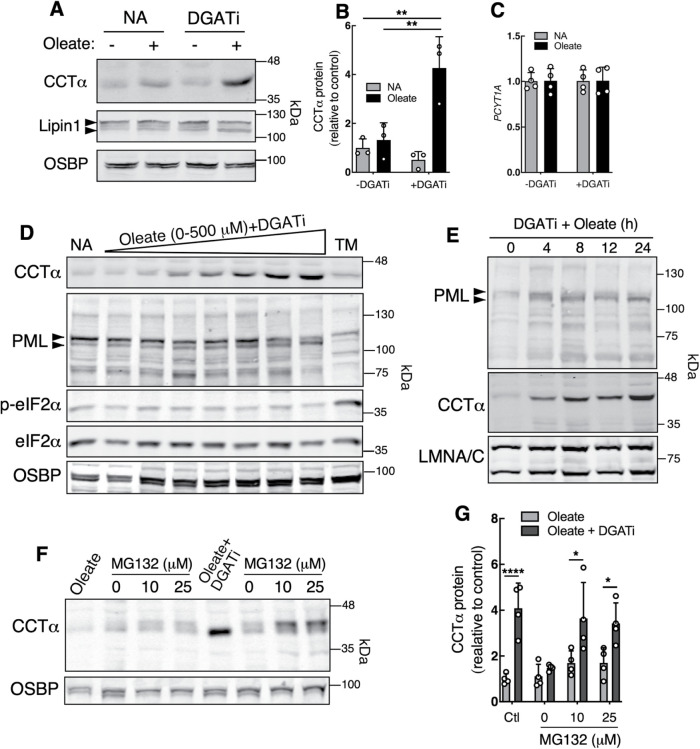
Stabilization of INM-associated CCTα in DGATi and oleate treated cells. (A) Lysates from U2OS cells treated with no addition (NA) or DGATi, with and without oleate for 16 h were immunoblotted for Lipin1, CCTα, and OSBP. (B) CCTα protein expression from panel A was quantified relative to no addition control (mean and SD of three experiments). (C) mRNA expression of *PCYT1A* in U2OS cells treated as described in panel A (mean and SD of four replicates from two experiments). (D) lysates of U2OS cells treated for 16 h with NA, DGATi (10 µM) in the presence of 0–500 µM oleate or tunicamycin (TM) were immunoblotted for CCTα, PML, eIF2α, pSer51 eIF2α (p-eIF2α) and OSBP (load control). (E) Immunoblots of lysates from cell treated with DGATi and oleate for 0 to 24 h. (F) U2OS cells were pretreated with oleate or oleate plus DGATi for 20 h followed by media without oleate or DGATi but supplemented with MG132 for 4 h. Cell lysates were immunoblotted for CCTα and OSBP. (G) Quantification of CCTα expression from panel H (mean and SD of four experiments) normalized to OSBP load control. Statistical significance was determined using Student's *t* test. *p<0.05, **p<0.01, ****p<0.0001.

Compared with controls, the fluorescence intensity of CCTα immunostaining on the INM and in the nucleoplasm of U2OS cells treated with DGATi and oleate was increased (e.g., Figure 2A and Supplemental Figure 2), indicative of increased CCTα protein expression. Indeed, immunoblotting of U2OS cells treated with DGATi plus oleate revealed a >four-fold increase in CCTα protein expression compared with control or oleate-treated cells (Figure 7, A and B). The expression of Lipin1 was not affected. The increase in CCTα protein was not due to elevated *PCYT1A* mRNA (Figure 7C). Analysis of other cell lines revealed variable effects of DGATi and oleate on CCTα expression. A lower molecular mass dephosphorylated isoform of CCTα was increased 2- to 3-fold in Huh7 and SH-SY5Y cells, respectively, treated with oleate plus DGATi (Supplemental Figure 4). In contrast, CCTα expression in F8 fibroblasts and HeLa cells was unaffected by the treatment (Supplemental Figure 4). In summary, U2OS, HeLa, SH-SY5Y, F8 and Huh7 cells displayed increased NE enrichment of CCTα following DGATi and oleate treatment (Figures 1 and 3; Supplemental Figure 2), but only in U2OS, Huh7 and SH-SY5Y cells was CCTα protein increased.

We further analyzed the mechanism for CCTα protein induction in U2OS cells. Treating U2OS cells with DGATi and increasing concentrations of oleate (0–500 µM) caused a progressive fourfold increase in CCTα protein expression as well as an increase in the mobility of a major 115 kDa PML isoform (Figure 7D). The ER stress response was not involved since the ratio of p-eIF2α/eIF2α, an indicator of PERK activation (Wang *et al.*, 2014), was unaffected by DGATi and oleate treatment compared with tunicamycin (Figure 7E). A time course of U2OS cells treated with oleate and DGATi revealed an increase in CCTα protein and a mass shift of the 115 kDa PML isoform as early as 4 h (Figure 7E). To identify the proteolytic system involved, U2OS cells were pretreated with oleate or oleate plus DGATi for 20 h, followed by replacement media lacking these additives but containing the proteosome inhibitor MG132 (Figure 7, F and G). The fourfold increase in CCTα expression in DGATi and oleate-treated cells returned to pretreatment levels in control replacement media. Replacement media containing MG132 prevented proteolytic degradation of CCTα, which appeared as a hyperphosphorylated higher molecular mass isoform. CCTα was reported to be mono-ubiquitinated, leading to retention in the cytoplasm and lysosomal degradation (Chen and Mallampalli, 2009). However, CCTα stability following DGATi and oleate treatment was not affected by the lysosomal inhibitor bafilomycin A1 (Supplemental Figure 5). These data imply that proteasomal degradation of CCTα occurs in the nucleoplasm after dissociation of the enzyme from the INM. As shown in Supplemental Figure 6, increased expression and NE localization of CCTα in DGATi and oleate-treated cells resulted in a four-fold increase in [^3^H]choline incorporation into CDP-choline and PC relative to oleate-treated cells. However, increased PC synthesis was countered by enhanced production of [^3^H]glycerophosphocholine, the product of phospholipase A hydrolysis of PC, in response to hyper-activation of CCTα at the INM.

### Correlation between DAG and PML patches in the INM

**FIGURE 8:**
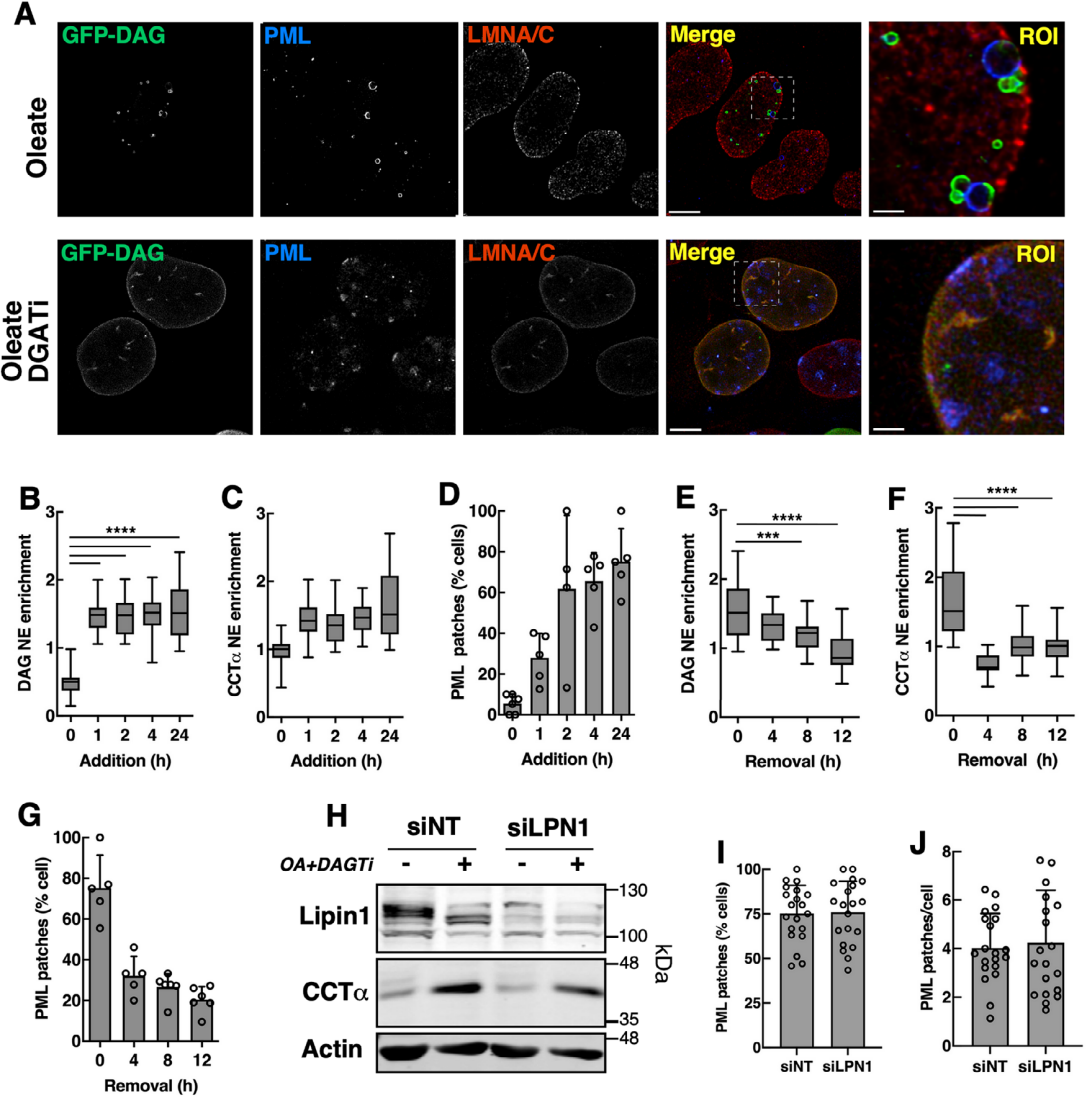
Temporal relationship between CCTα, PML and DAG content of the INM. (A) U2OS cells transiently expressing a nuclear-localized GFP-tagged DAG sensor and treated with oleate or oleate and DGATi for 24 h were immunostained for PML and LMNA/C (bar, 10 µm). A region of interest (ROI) from the merged images is shown (bar, 2 µm). (B, C, and D) Cells expressing the nGFP-DAG sensor were treated with oleate and DGATi for the indicted times and immunostained for PML. The NE enrichment of nGFP-DAG (B), NE enrichment of CCTα (panel C) and percentage of cells with PML patches (panel D) were quantified. (E, F, and G) Cells expressing nGFP-DAG sensor were pretreated with DGATi and oleate for 16 h followed by media with no addition. The NE enrichment of nGFP-DAG (E), NE enrichment of CCTα (F) and percentage of cells with PML patches (G) were quantified by confocal imaging at the indicated times. Results in panels B to G are the mean and SD from five fields of cells from representative experiments. (H) U2OS cells transfected with non-targeting siRNA (siNT) or siRNA targeting *LPIN1* (siLPN1) were treated with or without oleate plus DGATi for 24 h. Lysates were immunoblotted with antibodies against Lipin1, CCTα, and actin. (I and J) siNT and siLPN1 transfected cells treated with oleate plus DGATi were immunostained for PML along with BODIPY 493/503 and the percentage cells with PML patches (I) and PML patches per cell (J) was quantified. Results are from two representative experiments (22 fields of cells).

DAG and PML associate with nLDs (Lee *et al.*, 2020) and the INM of oleate and DGATi-treated Caco2 cells (McPhee et al., 2024b). To understand the relationship between PML patches and DAG-induced CES, we used a nuclear-localized sensor (Lee *et al.*, 2020) to correlate the appearance and disappearance of DAG with PML patches. The nGFP-DAG sensor was localized to the surface of nLDs in oleate-treated U2OS cells but did not overlap with PML staining on the surface of LAPS (Figure 8A). Inclusion of DGATi shifted the nGFP-DAG sensor to the INM, and nucleoplasmic puncta that co-localized with PML and LMNA/C (Figure 8A). nGFP-DAG was significantly enriched in the NE as early as 1 h after DGATi and oleate treatment (Figure 8B), as was CCTα (Figure 8C). This contrasts with a more gradual rise in the percentage of PML patch-positive cells (Figure 8D). Removal of DGATi and oleate following a 24 h treatment had little impact on INM-associated nGFP-DAG for the first 8 h (Figure 8E), but the NE enrichment of CCTα returned to control values within 1 h (Figure 8F), and the percentage of PML patch-positive cells fell from ∼75% to just over 30% in 4 h (Figure 8G). Lipin1 was silenced in U2OS cells with a small interfering RNA (siLPN1) in an attempt to inhibit DAG synthesis at the INM and block PML patch formation. However, a >80% knockdown of Lipin1 expression (Figure 8H) had no significant impact on the formation of PML patches in U2OS cells treated with DGATi and oleate (Figure 8I). CCTα localization to the INM was also unaffected by Lipin1 silencing (Figure 8J). Collectively, DGATi and oleate caused the appearance of both DAG and PML patches at the INM, but with different kinetics that could reflect the time required for PML patch assembly and disassembly and/or nGFP-DAG biosensor sensitivity.

### Alternate models of INM elastic curvature stress induce PML patches

**FIGURE 9:**
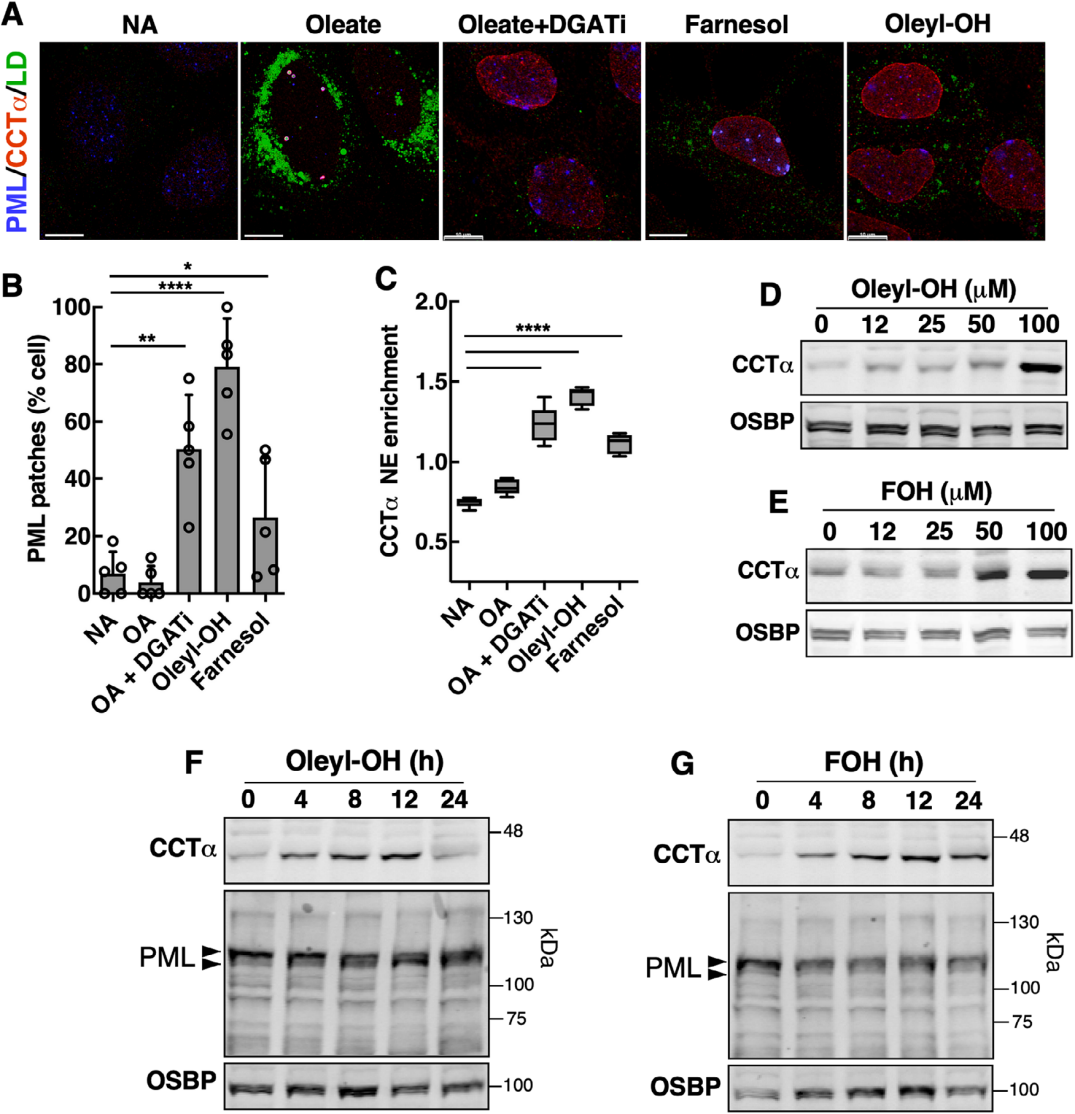
The CCTα activators oleoyl alcohol and farnesol induce PML patches. (A) U2OS cells treated with oleate and DGATi, 100 µM farnesol or 100 µM oleyl alcohol (Oleyl-OH) for 4 h were immunostained for PML and CCTα along with BODIPY 493/503 (bar, 10 µm). The percentage of cells with PML patches and (B) CCTα NE enrichment (C) was quantified. Results are the mean and SD of five fields of cells (*n* = 303) from a representative experiment. (D) U2OS cells treated with no addition (NA) or increasing concentrations of oleyl alcohol for 16 h were immunoblotted for CCTα and OSBP. (E) U2OS cells treated with NA or increasing concentrations of farnesol for 16 h were immunoblotted for CCTα and OSBP. (F) U2OS cells were treated with 100 µM oleyl alcohol for the indicated times and lysates were immunoblotted for PML, CCTα, and OSBP. (G) U2OS cells treated with NA or 100 µM farnesol for the indicated times and immunoblotted antibodies for PML, CCTα, and OSBP. Results for panels D–G were repeated three times with similar results. Statistical significance was determined using Student's *t* test. *p<0.05, **p<0.01, ****p< 0.0001.

Oleyl alcohol and the isoprenoid farnesol have small, neutral polar headgroups and bulky side chains that activate CCTα in liposome assays and promote CCTα translocation to the INM by inducing CES (Cornell and Vance, 1987; Lagace and Ridgway, 2005a). U2OS cells treated with oleyl alcohol or farnesol promoted CCTα localization and PML patches on the INM but did not increase cytoplasmic LDs or nLDs compared with oleate treatment (Figure 9A). A quantitative comparison showed that oleyl alcohol induced PML patches in 80% of cells compared with 25% and 50% in farnesol- and oleate plus DGATi-treated cells, respectively (Figure 9B). This relationship extended to NE enrichment of CCTα, which was slightly greater for oleyl alcohol compared with the other treatments (Figure 9C). Oleyl alcohol and farnesol also induced a dose-dependent increase in CCTα expression in U2OS cells at 16 h (Figure 9, D and E). A time course of oleyl alcohol and farnesol treatment indicated that increased CCTα expression was evident by 4 h and maximal by 12 h (Figure 9, F and G). The expression of PML isoforms was altered by oleyl alcohol treatment but not by farnesol (Figure 9, F and G). Thus, exogenous lipid activators of CCTα that induce CES at the INM also increase PML patches.

**FIGURE 10:**
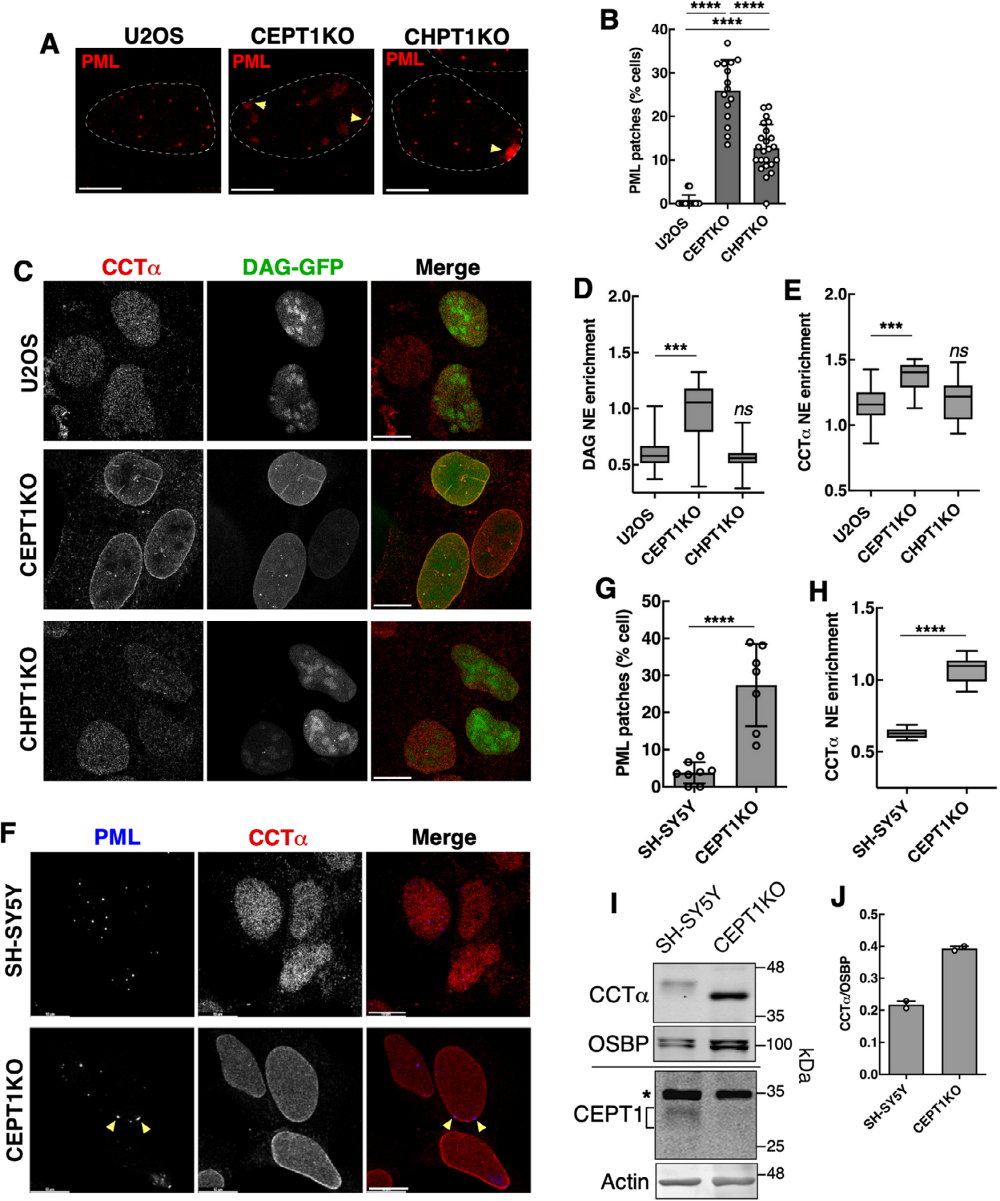
Knockout of the terminal enzymes in PC synthesis increases PML patch formation. (A) U2OS, CEPT1 KO, and CHPT1 KO cells were immunostained for PML. DNA was stained with Hoechst 33342 (bar, 5 µm). (B) The percentage of cells with PML patches was quantified from images in panel A and is the mean and SD from 12 fields of cells (*n* = 911) from three experiments. (C) U2OS, CEPT1 KO, and CHPT1 KO cells transiently expressing nGFP-DAG were immunostained for CCTα and LMNA/C (bar, 10 µm). (D and E) The NE enrichment of the nGFP-DAG (D) and CCTα (E; in GFP-positive nuclei) was quantified from two independent experiments. (F) control SH-SY5Y and SH-SY5Y CEPT1 KO cells were immunostained with antibodies against PML and CCTα (bar, 10 µm). (G and H) the percentage of cells with PML patches (G) and NE enrichment of CCTα (H) were quantified from eight fields of cells (*n* = 411) from a representative experiment. (I) Lysates from SH-SY5Y and SH-SY5Y CEPT1 KO cells were immunoblotted for CCTα and OSBP or CEPT1 and actin (asterisk indicates a non-specific band). (J) CCTα expression relative to OSBP was quantified from two experiments. Statistical significance determined by Student's *t* test; *** *p*<0.001, **** *p*<0.0001 (ns, not significant).

We previously reported that CRISPR knockout of CEPT1, the terminal enzyme in the CDP-choline pathway, increased PML patches and CCTα on the INM as well as CCTα protein expression (Dorighello *et al.*, 2023), a phenotype indicative of CES at the INM due to PC deficiency and/or increased DAG. We quantified the frequency of PML patches in U2OS and CEPT1 KO cells as well as U2OS cells with a CRISPR deletion of the choline phosphotransferase gene *CHTP1* (CHPT1 KO; Dorighello *et al.*, 2023). Approximately 25% of CEPT1KO cells were positive for PML patches compared with only 12% for CHPT1 KO cells (Figure 10A). The nGFP-DAG sensor (Figure 10, C and D) and CCTα (Figure 10, C and E) were significantly enriched on the INM of CEPT1 KO cells but not CHPT1 KO cells. We also analyzed SH-SY5Y cells with a CRISPR KO of CEPT1 to determine if PML patch formation occurred in another cell line lacking the terminal enzyme for PC and PE synthesis. PML patches were present in 25% of SH-SY5Y CEPT1 KO cells compared with <5% in controls (Figure 10, F and G). SH-SY5Y CEPT1 KO cells also had significant NE enrichment of CCTα (Figure 10H) and a twofold induction of CCTα protein expression (Figure 10, I and J). Thus, deficient PC synthesis in CEPT1 KO cells consistently enhanced PML patch formation, NE enrichment and expression of CCTα. Collectively, these data support a model wherein CES in the INM promotes PML-II membrane-associated patches.

## DISCUSSION

Fatty acid-induced stress causes the PML-II isoform to form patches on LAPS as well as the INM and Type-I NR (Ohsaki *et al.*, 2016; Soltysik *et al.*, 2019; McPhee *et al.*, 2024b). The association of PML-II with nLDs indicates an affinity for monolayers with gaps between phospholipid headgroups due to positive curvature and the presence of non-bilayer lipids. Using several cell models of membrane stress, we report a correlation between PML patches, CCTα, and DAG in the INM that indicates PML-II also associates with the INM under conditions of CES.

We initially observed that PML patches formed transiently at the INM of U2OS cells during the first hour of oleate treatment (Figure 1A). However, this did not coincide with the appearance of CCTα or DAG, indicating that PML patches are an early and sensitive indicator of INM stress that resolves when oleate and DAG are converted to TAG and packaged into nLDs. Preventing this resolution phase by inhibiting DGAT1 and two leads to constitutive INM stress and PML patches in response to a series of 18-carbon mono- and poly-unsaturated fatty acids, but less so for saturated species. Under these conditions, PML patches appeared in conjunction with the NE enrichment of CCTα and DAG, indicating CES due to increased DAG and/or incorporation of unsaturated fatty acids into phospholipids to increase acyl-chain volume relative to the head group. While the presence of DAG in the INM correlated with the formation and disappearance of PML patches (Figure 8), silencing of Lipin1 expression to reduce DAG levels had no effect on PML patches and NE enrichment of CCTα. This negative result is consistent with a report that Lipin1 silencing reduced NE enrichment of a DAG sensor by only 10%–15% (Lee *et al.*, 2023), possibly due to compensation by other Lipin isoforms or residual Lipin1 activity. As well, the DAG biosensor provides a qualitative estimate of membrane content that may not relate directly to its role in CES. Finally, DGATi and oleate treatment have the potential to elevate fatty acids and other fatty acid-derived lipids in the INM that could work in conjunction or independently of DAG to induce CES. In light of these ambiguities, the exact contribution of DAG to CES and PML recruitment to the INM is uncertain.

To confirm the role of CES in PML membrane association, two additional methods to induce lipid stress at the INM were used. Oleyl alcohol and farnesol activate CCTα when incorporated into cultured cells and liposomes (Lagace and Ridgway, 2005a), indicating they induce CES when incorporated into biological membranes. Similar to oleate-DGATi treatment, both oleyl alcohol and farnesol promoted PML patches, NE enrichment of CCTα and increased CCTα expression. A genetic approach was also used to increase CES at the INM, wherein CRISPR knockout of CEPT1 activity in U2OS and SH-SY5Y cells reduced PC synthesis in the ER from DAG, decreasing the ratio of the bilayer-to-non-bilayer lipids. Both CEPT1 KO cell lines had increased INM-localization and expression of CCTα accompanied by a 25%–30% increase in cells positive for PML patches. In contrast, knockout of Golgi-localized CHPT1 caused PML patches in only 12% of cells, and did not increase INM-associated CCTα and DAG. This discrepancy suggests that (1) blockage of PC synthesis in the ER specifically alters INM composition and activates CCTα, and (2) PML has a low threshold for sensing INM stress. Based on its close association with the CES sensor CCTα in different lipid stress models, we propose that PML-II also interacts with membranes that have gaps or voids in phospholipid packing due to the relative abundance of non-bilayer versus bilayer-forming lipids.

Structural and mutational analysis of PML-II has identified potential membrane interaction motifs in amino acids 571-882 encoded by the unique exon 7b as well as the conserved RBCC domain (Jul-Larsen *et al.*, 2010). PML-II patches in U2OS cells were reduced after deletion of amino acids 653-681 in exon 7b (Jul-Larsen *et al.*, 2010), a region that coincides with one of two potential 25- and 22-amino acid amphipathic helices identified using Alphafold2 (Janssen, 2025). Mutation of residues on the hydrophobic face of either helix, or an essential phenylalanine residue in the RBCC, reduced the frequency of PML-II patches in U2OS cells (Janssen, 2025). Thus, similar to CCTα, PML-II could interact with the INM by insertion of one or more amphipathic helices. The finding that PML-II patches are dependent on the RBCC domain further implies that the membrane affinity of PML-II monomers is relatively weak but enhanced by oligomerization. However, until the membrane binding properties of these helices are confirmed, the possibility that INM targeting is mediated by protein–protein interactions cannot be discounted.

PML patches induced by oleate and DGATi were deficient in the canonical PML NB proteins DAXX, SP100 and SUMO, a result also observed for LAPS (Lee *et al.*, 2020). In this respect, PML patches could be LAPS precursors that are unable to mature and release from the INM due to TAG deficiency. However, this is unlikely since PML patches were evident in DGATi and oleate-treated HeLa and SH-SY5Y cells that do not make nLDs or LAPS. Rather, PML patches form on the INM or nLDs due to recognition of CES in the planar INM or the positive curvature of the lipid droplet monolayer, respectively. PML NBs were significantly reduced by oleate and DGATi treatment, indicating they contribute to patch formation by direct recruitment to the INM or after disassembly into PML monomers that then multimerize at sites of membrane CES. Because PML patches are composed of PML-II, heterogeneous in size, and desumoylated, it is more likely they derive from soluble nucleoplasmic PML that is in equilibrium with PML NBs (Weidtkamp-Peters *et al.*, 2008). PML patches were also associated with regions of the NE that were deficient in LMNA/C and emerin (Figure 6; Jul-Larsen *et al.*, 2010), indicating a possible role in INM remodelling or repair. LMNA/C and emerin deficiency at PML patches indicates these are not sites of active repair after NE rupture (Young *et al.*, 2020; Kono *et al.*, 2022) but rather regions of the NE where sustained membrane stress induced stable PML recruitment and lamina depletion. In contrast, the transient appearance of PML patches in oleate-treated U2OS cells (Figure 1A) suggests these structures can rapidly resolve once membrane stress is relieved. Thus, PML-II could stabilize regions of CES until membrane phospholipid homeostasis was restored.

Membrane translocation and activation of CCTα on the INM also alleviates CES by increasing flux through the CDP-choline pathway and DAG conversion to PC. In addition, we observed that membrane CES in U2OS also increased CCTα expression four- to fivefold by protection from proteasomal degradation. CCTα stabilization occurred upon sustained translocation to the INM but not nLDs or LAPS and was cell type-dependent. Notably, in U2OS and HUH7 cells that assemble nLDs and LAPS, PML patch formation occurred in conjunction with elevated CCTα  NE enrichment and expression, whilst cells without nLDs or LAPS (HeLa, SH-SY5Y, and fibroblasts) showed a lack of correlation between PML patches and CCTα expression. Enzyme stabilization on the INM appears to be a mechanism to further enhance PC synthesis and restore membrane bilayer structure under conditions of sustained CES. In cells treated with DGATi and oleate (Supplemental Figure 5), oleyl alcohol or farnesol (Lagace and Ridgway, 2005a), elevated PC synthesis was counter-balanced by degradation to GPC as a result of sustained CCTα activation at the INM and uncoupling of feedback regulation.

Despite being relatively planar, the INM is subject to cytoplasmic and nuclear forces, and localized glycerolipid metabolism that cause dynamic changes in membrane composition and structure. We have extended previous studies on the INM composition and nuclear protein recruitment (Cornell and Ridgway, 2015; Bahmanyar and Schlieker, 2020; Lee *et al.*, 2023) to show that membrane CES also promotes the disassembly of phase-separated PML NBs into INM-associated patches. Since there is a basal level of PML patches (Figure 7; Jul-Larsen *et al.*, 2010), and patches on nucleoplasmic reticulum occur during the production of nLDs and LAPS in Huh7 cells (Soltysik *et al.*, 2019), they could be important in responding to localized CES at the INM. PML patches occurred in conjunction with CCTα activation on the INM, which drives PC synthesis to restore planar bilayer structure. This combined response of PML-II and CCTα to aberrant INM composition could protect the integrity of the nucleus by restoring membrane homeostasis.

## MATERIALS AND METHODS

Request a protocol through *Bio-protocol*

### Cell culture and transfection

U2OS (ATCC HTB-96), U2OS PML knockout (KO; Attwood *et al.*, 2019), U2OS CEPT1 KO (Dorighello *et al.*, 2023), U2OS CHPT1 KO (Dorighello *et al.*, 2023), U2OS Clover-PML knock-in (Pinder *et al.*, 2015), SH-SY5Y, SH-SY5Y CEPT KO, F8 human skin fibroblasts and Huh7 (JCRB0403, Japanese Collection of Research BioSources Cell Bank) cells were cultured in DMEM containing FBS (10%; medium A) at 37°C in a CO_2_ (5%) atmosphere. CRISPR/Cas9 editing was used to knock out *CEPT1* in SH-SY5Y cells using guide RNAs targeting exon 2; chr1(GRCh38):g.111147741-111147760 (5′-AAGATGTGGAGATTCTCACC-3′) and chr1(GRCh38):g. 111,147,970-111,147,989 (5′-AATCTCATCACCATCATTGG-3′; Ran *et al.*, 2013). gRNAs were cloned into either pSpCas9(BB)-2A-GFP (PX458, Addgene: #48138) or pU6-(BbsI) CBh-Cas9-T2A-mCherry (Addgene: #64324). Fluorescent clonal cell lines were isolated and screened for disruption of *CEPT1* by PCR and sequencing. Western blotting confirmed the absence of CEPT1 protein expression (Figure 10I).

Cells were incubated with palmitate, oleate, linoleate or linolenate complexed with BSA (6.6:1 mol/mol). Stock solutions of inhibitors of DGAT1 (A922500; Sigma-Aldrich), DGAT2 (PF-06424439; Sigma-Aldrich), and SUMO activating enzyme (ML-792; Selleckchem) were prepared in DMSO. Stock solutions of oleyl alcohol (Sigma-Aldrich) and farnesol (Sigma-Aldrich) were prepared in ethanol. Tunicamycin (Sigma-Aldrich), bafilomycin A1 and MG132 (Sigma-Aldrich) stock solutions were prepared in DMSO.

U2OS cells seeded on glass coverslips were transiently transfected with plasmids encoding GFP-PML isoforms or the DAG sensor GFP-C1(2)δ-2xNLS (nGFP-DAG; Addgene #21216) using Lipofectamine 2000 (ThermoFisher) at a ratio of 3:1 (µg plasmid/µl Lipofectamine) for 48 h. Lipin1 expression in U2OS cells was silenced by transient transfection of siLPIN1 or a non-targeting siNT (200 nM; Horizon Discovery, ONTARGETPlus SMARTPool) with Lipofectamine 2000 for 48 h.

### Immunostaining and confocal immunofluorescence microscopy

Cells cultured on glass coverslips were fixed with paraformaldehyde (4%, wt/vol) for 15 min and permeabilized with Triton X-100 (0.2%, wt/vol) in PBS for 10 min at 4°C. Coverslips were incubated at 4°C for 24 h in PBS containing BSA (1%, wt/vol) with antibodies against PML (Santa Cruz, sc-377390; Bethyl, A301-167A), LMNA/C (Cell Signaling, 4777), CCTα (Lagace and Ridgway, 2005b), emerin (sc15378; Santa Cruz), DAXX (D7810; Sigma-Aldrich), SP100 (PA5-53602; Invitrogen), or SUMO (monoclonal Y299 Ab32058; Abcam). Following incubation with AlexaFluor-647 and AlexaFluor-555 secondary antibodies for 1 h, LDs were stained with BODIPY 493/503 or LipidTox Red in PBS for 30 min at 20°C. DNA was stained with Hoechst 33342, and coverslips were mounted on slides in MOWIOL 4-88. Cells were imaged using a Leica TCS SP8 near super-resolution confocal microscope with 4 solid state lasers (405 nm, 488 nm, 552 nm, and 638 nm), HC Plan APOCHROMAT CS2 63X/1.4 numerical aperture lens, and LASX software set to the Lightning mode. Identical channel settings were used to capture images for control and treated cells.

LAPS, PML patches, and PML-NBs in confocal sections of cells (0.8–1 µm) were quantified using Image J (v1.53) as previously described but with the addition of binary masks for the nucleus defined by Hoechst staining and PML (McPhee et al., 2024b). To quantify the percentage of cells with LAPS in U2OS and Huh7 cells, the nuclei and overlapping BODIPY and PML structures were quantified using the cell counter plugin in Image J (v1.53). The number of PML patches on the NE was quantified from confocal images of cells immunostained for PML together with BODIPY and Hoechst staining. The number of nuclei with 1 or more PML patches on the nuclear periphery (defined by Hoechst or LMNA/C staining) or the total number of patches per nucleus devoid of BODIPY staining were manually counted using ImageJ software (v1.53). Finally, PML NBs in the nucleoplasm that were devoid of BODIPY staining were quantified by taking the total number of PML structures per field of cells and subtracting the number of LAPS and/or PML patches.

The relative enrichment of SUMO in PML structures was quantified as follows. U2OS cells treated with no addition, oleate, DGATi plus oleate, or ML-792 for 24 h were fixed and immunostained with PML and SUMO antibodies, followed by Hoechst and LD staining. Confocal images were imported into ImageJ (v1.53), and a nuclear mask from the Hoechst channel was used to measure the mean intensity value of all nuclei per field of cells in the SUMO channel (total SUMO value). A mask from the PML channel was used to measure the mean intensity value for SUMO on all PML objects per field of cells (PML SUMO value). The SUMO enrichment score was calculated as a ratio of PML SUMO to total SUMO.

### Quantitation of nGFP-DAG and CCTα enrichment of the NE

U2OS, U2OS CEPT KO, and U2OS CHPT1 KO cells transiently expressing nGFP-DAG were immunostained for LMNA/C, and either PML or CCTα, the nuclei were stained with Hoechst, and imaged by confocal microscopy. NE enrichment of nDAG-GFP was quantified using ImageJ (v1.53). Nuclei of GFP-positive cells were cropped individually, and a total nuclear mask was generated using the ImageJ wand tool. Mean fluorescence intensity of nuclei was measured to ensure that each treatment group had a similar range of nGFP-DAG expression. The selection size was reduced by 9 pixels before measuring the mean fluorescence intensity of the nucleoplasm, which was then used to remove the nucleoplasmic area from the total nuclear mask and generate a selection area containing only the NE. The ratio of the mean fluorescence intensities of the nuclear periphery to the nucleoplasm provided a NE enrichment value. The same approach was used to quantify CCTα NE enrichment in the cropped nuclei of nGFP-DAG-transfected cells.

### SDS–PAGE and immunoblotting

Cells were lysed in 2xSDS lysis buffer (12.5% [wt/vol] SDS, 30 mM Tris-HCl [pH 6.8], 12.5% [vol/vol] glycerol, 0.01% [wt/vol] bromophenol blue, and 2% [vol/vol] β-mercaptoethanol), heated at 95°C for 3 min, sonicated for 10 s and resolved by SDS–PAGE. After transfer, nitrocellulose membranes were incubated in LI-COR Odyssey blocking buffer diluted 5:1 (vol/vol) with TBS-Tween (20 mM Tris-HCl [pH 7.4], 150 mM NaCl and 0.1% Tween 20) containing primary antibodies described in the previous section including those against β-actin (A5441; Sigma Aldrich), OSBP (Lagace *et al.*, 1999) and CEPT1 (N-14; Santa Cruz). This was followed by goat anti-rabbit or goat anti-mouse IRDye 800CW and/or IRDye 680LT-labelled secondary antibodies (LI-COR Biosciences). Membranes were scanned using a LI-COR Odyssey imaging system, and fluorescence intensity was quantified using associated software (v3.0).

### Quantification of [^3^H]oleate incorporation into neutral and polar lipids

U2OS cells received no addition, iDGAT1 (10 µM), iDGAT2 (10 µM), or a combination of both inhibitors (DGATi, 10 µM each) for 1 h before replacing media with the same treatments supplemented with [^3^H]oleate/BSA (100 µM, 6.44 µCi/ml) for 4 h. Following treatments, cells were rinsed twice with TBS containing BSA (2% wt/vol) and once with TBS. Total lipids were extracted from dishes with hexane:isopropanol (3:2, vol/vol), dried under N_2_ and resolved by thin-layer chromatography (TLC) using hexane:diethyl ether:acetic acid (90:30:1, vol/vol). TAG, cholesterol esters, DAG, and polar lipids (identified by standards) were scraped from the TLC plate, and [^3^H]oleate incorporation was quantified by liquid scintillation counting and expressed relative to total cellular protein.

### CDP-choline pathway activity measured by [^3^H]choline labelling

U2OS cells received no addition, oleate (500 µM) or DGATi for 1 h followed by replacement with choline-free media containing [^3^H]choline (1 µCi/ml) and the same additives. Cells were harvested in methanol:water (5:4, vol/vol) at the indicated times, and PC and water-soluble metabolites (phosphocholine, CDP-choline, and glycerophosphocholine) were extracted, separated and quantified by liquid scintillation counting (Storey *et al.*, 1997). [^3^H]Choline incorporation was expressed relative to total cellular protein.

### Quantitative PCR

*PCYT1A* and *PCYT1B* transcript levels in U2OS cells were measured by qPCR. Briefly, single-strand cDNA was prepared from total RNA (QIAGEN RNeasy Mini Kit) using SuperScript II Reverse Transcriptase and an oligo(dT)12-18 primer mix (Invitrogen, catalogue: 18418012). Samples containing cDNAs (100 ng), primer sets for *PCYT1A*, *PCYT1B*, and *PGK1*, and SYBR Green PCR Master Mix were analyzed on a BioRad CFX Opus 384 Real-Time PCR system for 30 cycles. The expression of *PCYT1A* and *PCYT1B* relative to *PGK1* was determined by the ΔΔCT method.

### Statistical analysis

Statistical analyses were performed using GraphPad Prism software (v.6.0) as indicated in figure legends. Two-way ANOVA analyses were corrected for multiple comparisons using Tukey's test.

## Supporting information





## Abbreviations used:

CES: curvature elastic stress
DAG: diacylglycerol
DGAT: diacylglycerol acyltransferase
INM: inner nuclear membrane
LAPS: lipid-associated PML structures
ONM: outer nuclear membrane
NE: nuclear envelope
nLD: nuclear lipid droplet
NPC: nuclear pore complex
PML NB: promyelocytic leukemia nuclear body.
