# Supplementary material for: Membrane curvature elastic stress triggers recruitment of PML-II onto the inner nuclear membrane
Source: Mol Biol Cell. 2025 Dec 10;37(2):ar10. doi: 10.1091/mbc.E25-09-0443 (PMC12879034; doi:10.1091/mbc.E25-09-0443)
Supplement: Supplementary file 1 [file mbc-37-ar10-s001.pdf]

Supplemental Materials

Molecular Biology of the Cell

McPhee *et al*.

Supplemental Material

McPhee et.al. Membrane curvature elastic stress triggers recruitment of PML-II onto the inner nuclear membrane

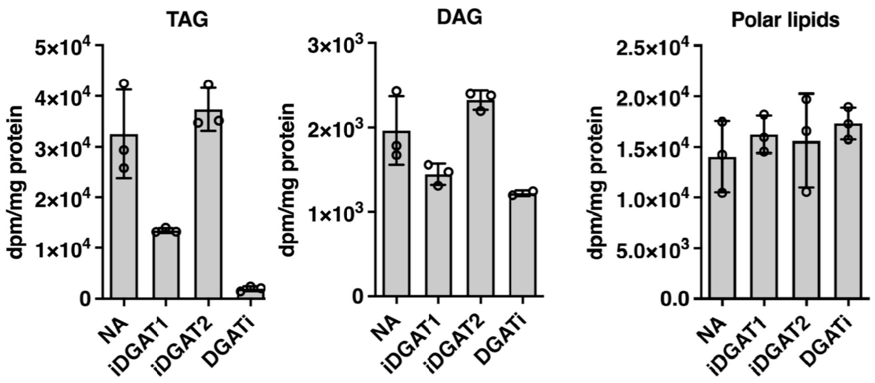

Supplemental Figure 1. Inhibition of triglyceride synthesis by DGAT1 and DGAT2 inhibitors. U2OS cells were treated for 4 h with [^{3}H]oleate in media containing no addition (NA), iDGAT1 (10 µM), iDGAT2 (10 µM), or a combination of both iDGAT1 and iDGAT2 (DGATi; 10 µM each). [^{3}H]Oleate incorporation into TAG, DAG and polar lipids is the mean and SD of three technical replicates from a representative experiment.

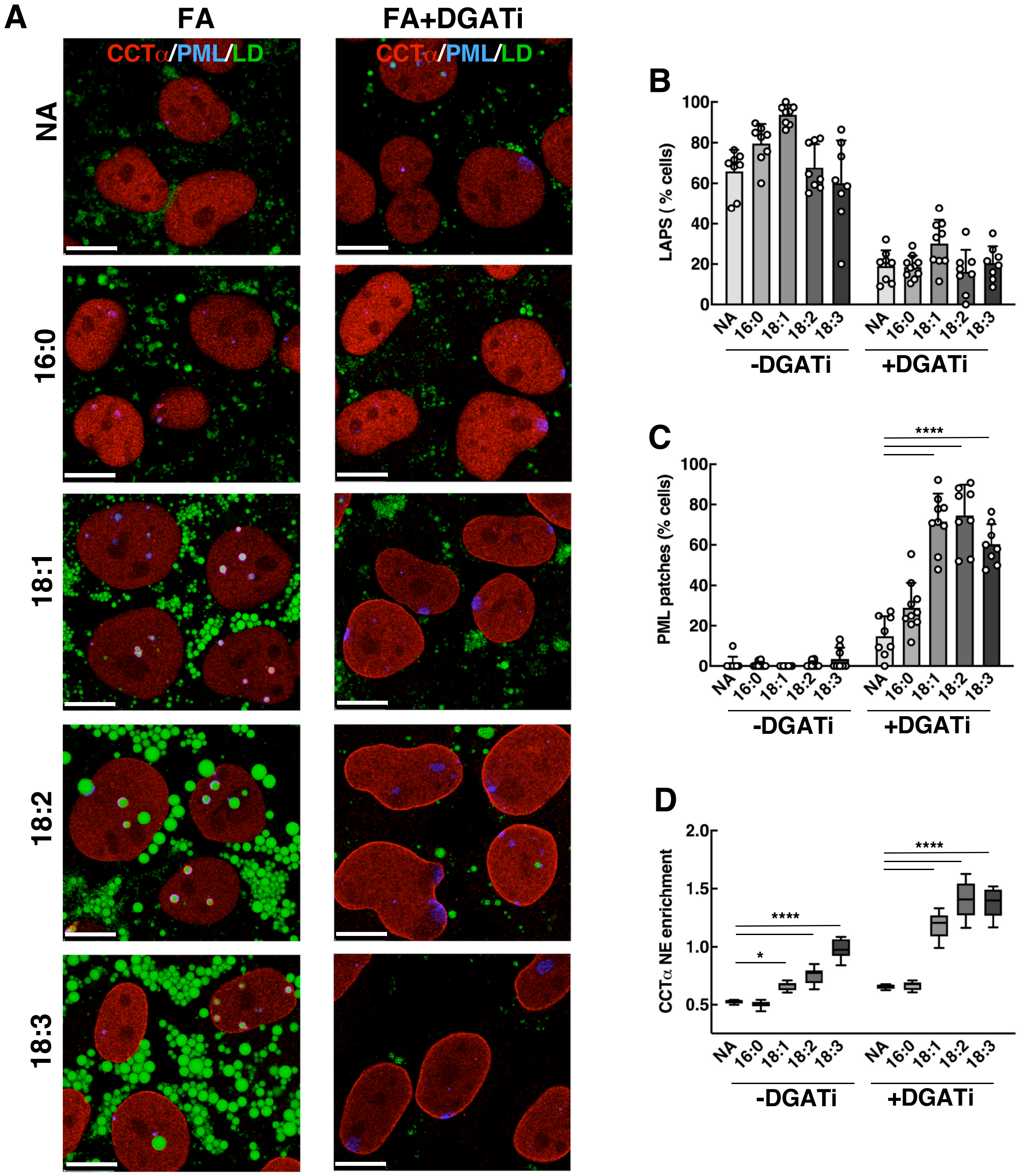

Supplemental Figure 2. INM localization of CCTα and PML patches in Huh7 cells treated with unsaturated fatty acid and DGATi. (A) Huh7 cells cultured in media with no addition (NA), palmitate, oleate, linoleate or linolenate, with or without DGATi, for 24 h were immunostained for PML and CCTα, and LDs were visualized with BODIPY 493/503 (bar, 10 μm). (B) The percentage of cells with LAPS. (C) Percentage of cell containing PML patches. (D) NE enrichment of CCTα. Results are from 8 fields of cells (n = 2261) from a representative experiment. Statistical significance was determined using two-way ANOVA. $^*p< 0.05$, $^{***}p<0.001$, $^{****}p< 0.0001$.

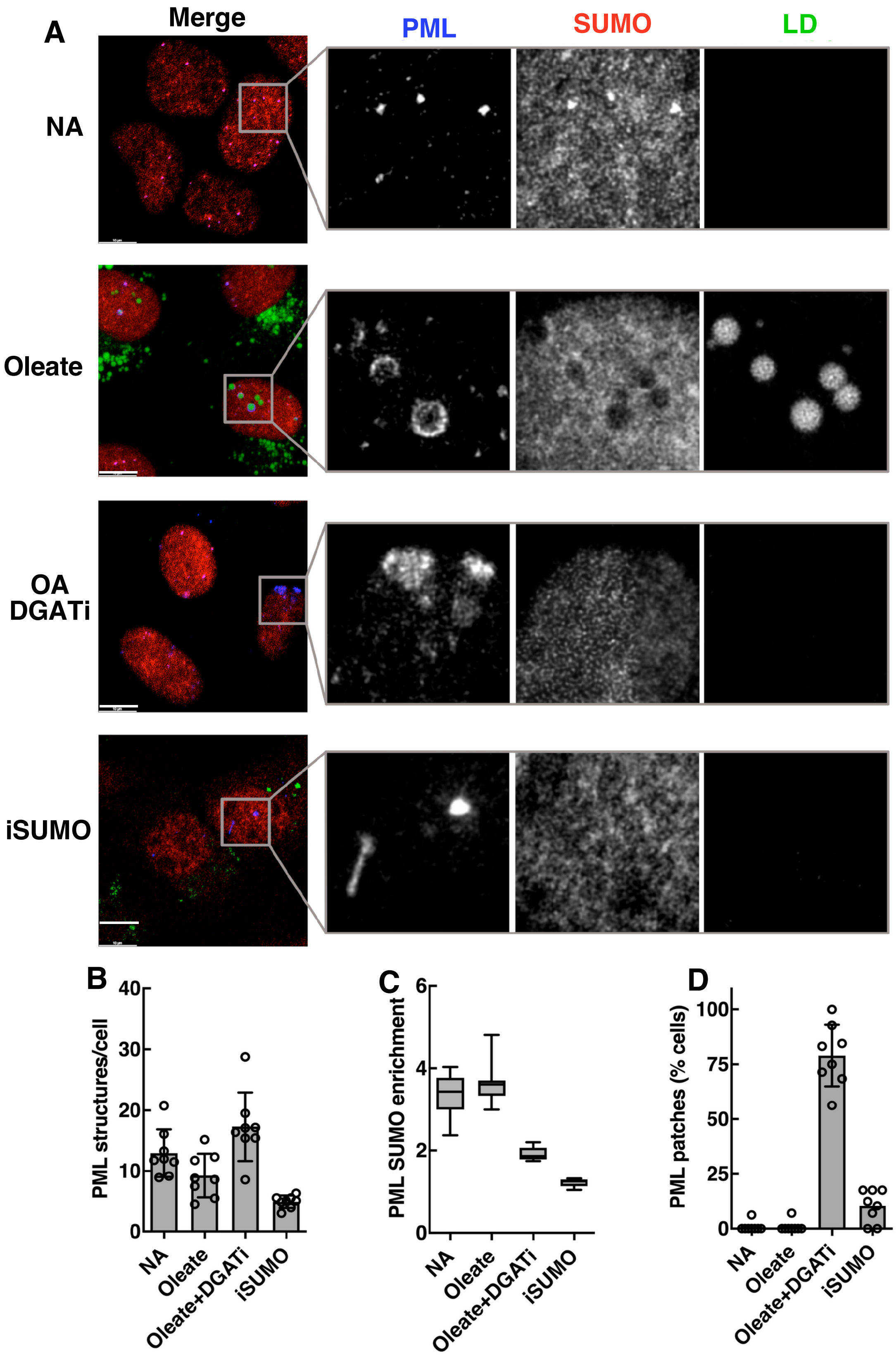

Supplemental Figure 3. DeSUMOylation of PML does not promote patch formation. (A) U2OS cells treated with no addition (NA), oleate, DGATi and oleate, or iSUMO (5 μM) for 24 h were immunostained for PML and SUMO while LDs were detected with BODIPY 493/503 (bar, 10 μm). (B) Total number of PML structures per cell. (C) Enrichment of SUMO in PML structures. (D) Percentage of cells with PML patches. Results in B, C, and D are the mean and SD of 8 fields of cells (n = 502) from a representative experiment.

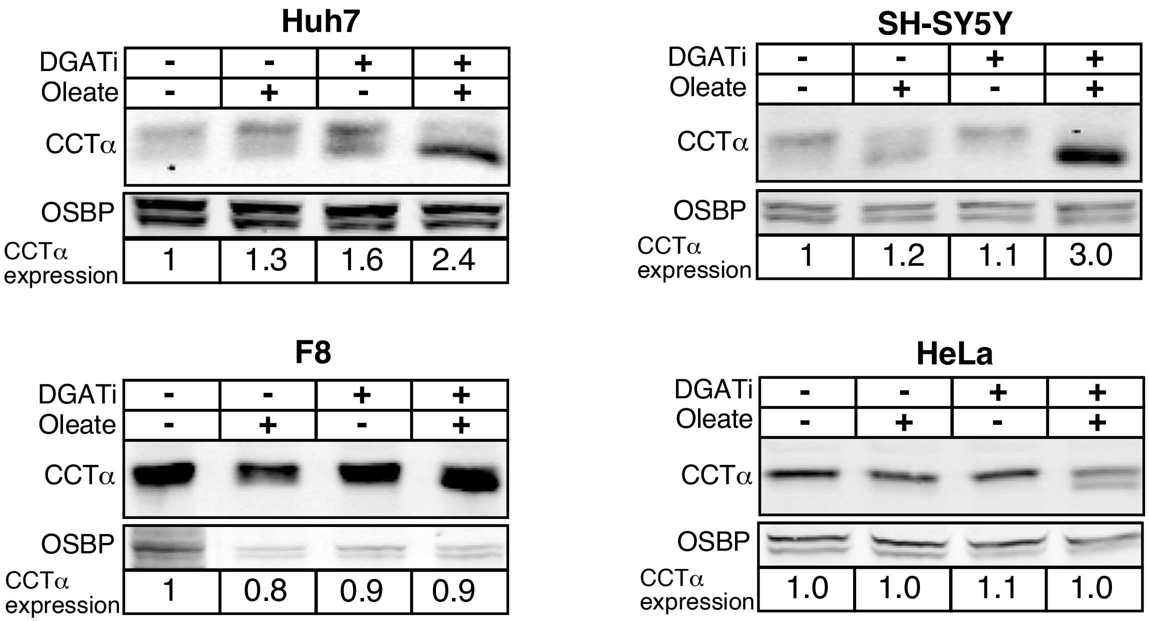

Supplemental Figure 4. CCTα induction by oleate and DGATi in cells lacking nLDs. Whole cell lysates of Huh7, SH-SY5Y, F8, and HeLa cells treated with combinations of oleate (500 μM) and DGATi (10 μM each) for 16 h were immunoblotted for CCTα and OSBP. CCTα expression was normalized to OSBP and expressed relative to untreated cells. Results for Huh7 and SH-SY5Y cells are from two independent experiments done in duplicate, while results for the other three cell lines are representative experiments.

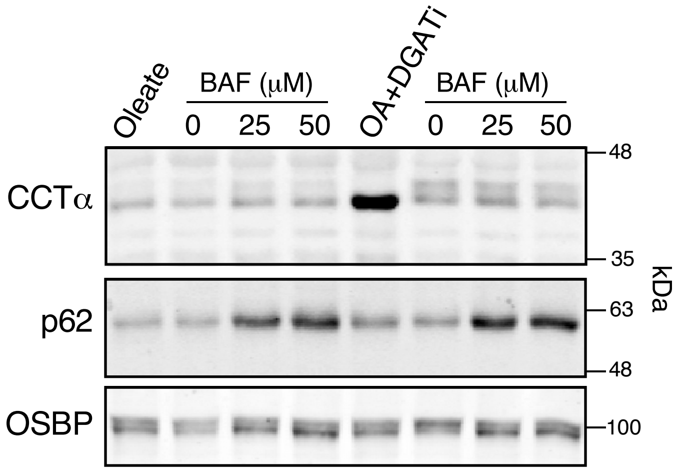

Supplemental Figure 5. Lysosomal inhibition does not effect CCTα stability. U2OS cells were pre-treated with oleate plus/minus DGATi for 20 h prior to replacing with media supplemented with Bafilomycin A1 (BafA1; 0, 24, 50 μM) for 4 h. Lysates were immunoblotted with antibodies against p62, CCTα, and OSBP. Results are from a representitive experiment.

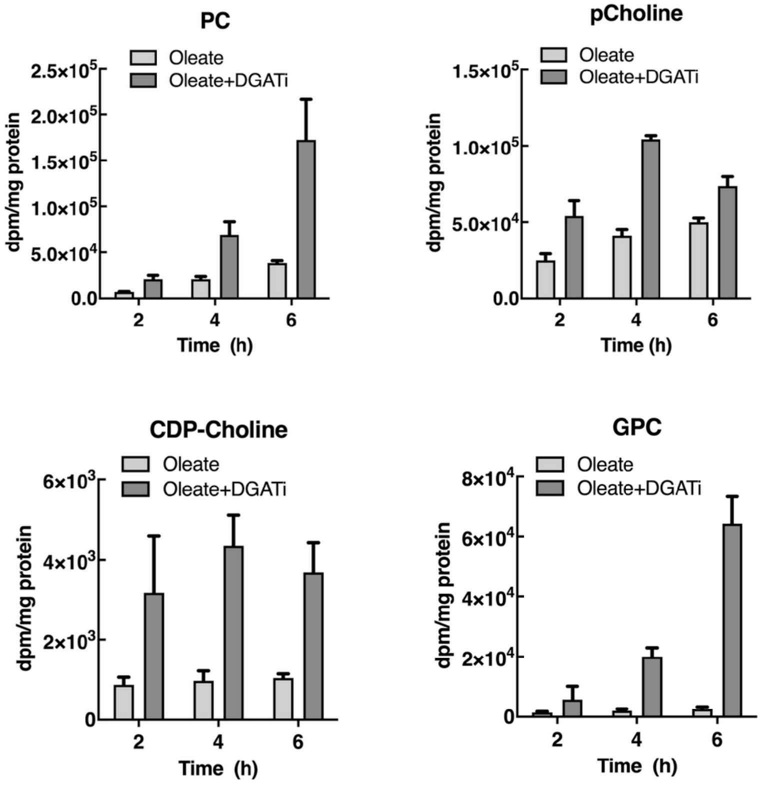

Supplemental Figure 6. Increased PC synthesis and degradation in oleate and DGATi-treated U2OS cells. U2OS cells treated with oleate (500 μM) or oleate plus DGATi (10 μM) were pulse-labelled with [^{3}H]choline (1 μCi) for the indicated times. [^{3}H]Choline incorporation into PC and water-soluble choline metabolites was quantified relative to total cell protein. Results are the mean and SD of triplicate determinations from a representative experiment.
