# Supplementary material for: Membrane curvature elastic stress triggers recruitment of PML-II onto the inner nuclear membrane
Source: Mol Biol Cell. 2025 Dec 10;37(2):ar10. doi: 10.1091/mbc.E25-09-0443 (PMC12879034; doi:10.1091/mbc.E25-09-0443)
Supplement: Supplementary file 2 [file reviewer_reportE25-09-0443.pdf]

Membrane curvature elastic stress triggers recruitment of PML-II onto the inner nuclear membrane

Michael McPhee, Jayme Salsman, Allison Newman, Nikol Voutsina, Andrew Crosby, Graham Dellaire, and Neale Ridgway

Corresponding author(s): Neale Ridgway, Dalhousie University

Review Timeline:

Submission Date:	2025-09-19
Editorial Decision:	2025-10-16
Revision Received:	2025-11-04
Accepted:	2025-12-02

Editor-in-Chief: John Aitchison

Transaction Report:

(Note: With the exception of the correction of typographical or spelling errors that could be a source of ambiguity, letters and reports are not edited. The original formatting of letters and referee reports may not be reflected in this compilation.)

RE: Manuscript #E25-09-0443
TITLE: Membrane curvature elastic stress triggers recruitment of PML-II onto the inner nuclear membrane

Dear Neale,

Thank you for submitting your paper entitled " Membrane curvature elastic stress triggers recruitment of PML-II onto the inner nuclear membrane " to MBoC. Two experts in the field have now evaluated your manuscript. The comments of these Reviewers are generally positive, and the significance of your findings to the nuclear envelope field are appreciated. However, they have raised various issues that need to be addressed. Both Reviewers pointed out several instances where conclusions made in the manuscript were not sufficiently supported by your results. Moreover, they shared concerns that some observations failed to add to the overall narrative of the manuscript (e.g. see Reviewer 1's comments on CCTa expression and NE enrichment) or were incomplete (e.g. see Reviewer 2's point 7). I would ask that you address the concerns of the reviewers in a revised version of your manuscript and submit a point-by-point response to the Reviewers' comments, indicating how you have incorporated your responses into the revised manuscript. Also, please note that MBoC strongly discourages authors from referencing 'data (or results) not shown'. Rather, supportive data that is not critical to the major finding can be published online as Supplemental Material.

Best regards,

Rick

Richard Wozniak
Monitoring Editor
Molecular Biology of the Cell

Dear Dr. Ridgway,

The review of your manuscript, referenced above, is now complete. The Monitoring Editor has decided that your manuscript is not acceptable for publication at this time, but may be deemed acceptable after specific revisions are made, as described in the Monitoring Editor's decision letter above and the reviewer comments below.

A reminder: Please do not contact the Monitoring Editor directly regarding your manuscript. If you have any questions regarding the review process or the decision, please contact the MBoC Editorial Office (mboc@ascb.org).

When submitting your revision include a rebuttal letter that details, point-by-point, how the Monitoring Editor's and reviewers' comments have been addressed. (The file type for this letter must be "rebuttal letter"; do not include your response to the Monitoring Editor and reviewers in a "cover letter.") Please bear in mind that your rebuttal letter will be published with your paper if it is accepted, unless you have opted out of publishing the review history.

Authors are allowed 90 days to submit a revision. If this time period is inadequate, please contact us at mboc@ascb.org.

Revised manuscripts are assigned to the original Monitoring Editor whenever possible. However, special circumstances may preclude this. Also, revised manuscripts are often sent out for re-review, usually to the original reviewers when possible. The Monitoring Editor may solicit additional reviews if it is deemed necessary to render a completely informed decision.

In preparing your revised manuscript, please follow the instruction in the Information for Authors (www.molbiolcell.org/info-for-authors). In particular, to prepare for the possible acceptance of your revised manuscript, submit final, publication-quality figures with your revision as described.

To submit the rebuttal letter, revised manuscript, and figures, use this link: Link Not Available

MBoC offers the option to publish your work with immediate open access. Open access can increase the discoverability and usability of your research. If you would like to publish your paper with immediate open access but did not select this option during initial submission, please contact the editorial office at mbc@ascb.org.

Authors of Articles and Brief Communications are also encouraged to create a short video abstract to accompany their article when it is published. These video abstracts, known as Science Sketches, are up to 2 minutes long and will be published on

YouTube and then embedded in the article abstract. Science Sketch Editors on the MBoC Editorial Board will guide you as you prepare your video. Information about how to prepare and submit a video abstract is available at www.molbiolcell.org/science-sketches. Please contact mboc@ascb.org if you are interested in creating a Science Sketch.

Sign up for MBoC's new content alerts here https://tinyurl.com/3bcsyk3e

Thank you for submitting your manuscript to MBoC. We look forward to receiving your revised paper.

Sincerely,

Eric Baker
Journal Production Manager
MBoC Editorial Office
mbc@ascb.org

Reviewer #1 (Remarks to the Author):

See attached.

Reviewer #2 (Remarks to the Author):

PML-II is a component of PML nuclear bodies, and prior work has shown that PML-II forms patches on the INM as well as the surface of nLDs under certain metabolic conditions. PML NBs recruit client proteins associated with several pathways, including DNA repair, which may be regulated through this relocation. This manuscript addresses whether PML-II associates with the inner nuclear membrane in response to lipid stress and/or an associated change in INM lipid composition. The authors show that PML patches at the INM form in the presence of oleate and inhibition of DGATs as well as PC deficiency and non-bilayer modification, and that this is distinct from PML association on the surface of nLDs that occurs in the presence of oleate. PML patches are present concurrently with the enrichment of CCTa and an INM DAG sensor leading them to conclude that curvature elastic stress triggers PML reorganization to these sites. Most of their experiments are done in U2OS cells, but they also test other cell types. Interestingly, the INM is remodeled at sites of PML patches. They go on to show that CCTa protein levels depend on the proteasome and that it is stabilized and at higher steady state levels with oleate + DGATi. Their data showing that knockout of the choline-ethanolamine transferase (CEPT1) that results in PC deficiency, results in INM DAG and CCTa localization as well as PMLII patches.
This paper shows convincing correlative data that INM recruitment of CCTa under most lipid stress conditions is concomitant with PML II patch formation. The authors use orthogonal approaches to support their interpretations of lipid stress inducing PML patch formation and INM-association of CCTa, which is the strength of the paper. Their observations for PML II patch formation corresponds INM reorganization at those sites is interesting, and might be an indication of abnormal nuclear envelope stability; however there paper falls short on making such a claim (see below). Overall, the data is for the most part convincing, yet the paper is a bit difficult to read through as they sometimes leave out conclusions from their results or in some cases they overinterpret their results (including in the significance statement). In addition, the middle of the paper presents several less critical and flushed out pieces of data and, at times, it becomes difficult to gain clarity of how the authors reach their big picture conclusions, and which results are most important to making the conclusions. My suggestion is for the authors to consolidate some of the data that is less important, and focus on the most important experimental results to reach their claim. Below are some detailed suggestions.
Major points:
1. How do the authors count the percentage of cell with LAPS? Are only cells that have nLDs counted? Do most cells contain nLDs? This information would help interpret Figure 2 B, which shows that on average there are 75% of cells have LAPS in the 18:1 treatment. Are PML patches counted in the same way as cells with LAPS?
2. A better description of how the authors distinguish between PML patches and LAPS in main text would be helpful. Sometimes they use co-markers like nLD markers in Figure 1D and Figure 2, but Fig. 1A where they do a lot of there quantitation does not have an nLD marker. It seems as though it may be hard in this case to distinguish between LAPS versus patches in some cases.
3. Figure 3 is where the paper begins to present a number of smaller points that don't always seem to be critical to the big picture question. One way to tie in the results from figure 3 is to provide the readers with some context on why the different cell lines in particular were chosen? What might be different about these cell types that elicit different effects on PML patches? Interestingly, fibroblasts relocate CCTa very robustly, suggesting INM lipid stress, yet they do not form PML patches. Could it be that these cells don't form nLDs? These results might tell us something about why some cell types make nLDs while others don't? For example, what about localization of DGATs or seipin in these cells? Or what about the make up of PML patches themselves? Are PML NBs very different in composition in the cell types that don't have it? While the authors don't have to perform more experiments, they should frame the results and the rationale more clearly to provide the reader with some clarity

to help with their understanding of the experiments and data in the context of the questions.
4. The authors used endogenously tagged Clover - PML to determine the localization of other factors to PML patches in Fig. 5. The lamin A/C clearance is obvious in some cells in top panel of Fig. 5A; however, lamin A/C staining itself looks a bit abnormal and there is no clear rim in the ROI magnification in the bottom panel of Fig5A. Is it possible that the authors did a max projection for the magnified images? It is difficult to see a clear rim in these zoom images, even though the ROI was taken from a region with a clear rim.
5. Note that although the clearances of laminA/C and somewhat of Emerin are clear, ruptures normally accumulate LaminA/C and Emerin (PMCID: PMC9617480, PMCID: PMC4833568, PMCID: PMC7521799 etc.). This is because they are involved in the repair process. Thus, clearance of lamin B1 is a good indicator of rupture sites, but the authors do not stain for lamin B1. It is possible that the lack of lamin A/C and emerin are because those sites have issues with repair or are large and so there is slow repair. However, the authors would have to do more work to test these possibilities. It is more likely at this point that these regions are remodeled or reorganized INM sites (perhaps more prone to rupture, but the paper does not really test this). To test if there are indeed more ruptures under this condition, live cell experiments with fluorescent reporters such as GFP:NLS or a fluorescent fusion to cGAS are needed. Furthermore, EM would help to visualize whether the nuclear membrane near these patches is intact or defective. These questions are interesting and important, but not fully addressed in the paper as is. The authors should change their significance statement to reflect this.
6. The result in Fig. 6 is confusing because the authors are quantifying LAPS in the Fig. 6A with Oleate treatment yet there are no nLDs in nuclei of the representative cells expressing GFP-PMLI and GFP-PML-IV. Does the expression of these fusion proteins prevent nLDs? Similar to the comment regarding Fig. 1, it is important to specify the percentage of cells with nLDs so we can understand the data better. Even the data with the GFP fusion construct looks different in Fig. 6 when compared with the quantitation performed with immunostaining in the earlier figures. This suggests that the GFP fusion (and perhaps the clover fusion from Fig. 5) may not be fully functional. This is important to discuss for the results of this figure.
7. Does the depletion of lipin1 affect GFP-DAG localization to the INM? Fig. 8I, J are difficult experiment to interpret without some internal controls for DAG levels. There may be no impact on PML patch formation because of compensatory mechanisms to generate DAG, for example. To add to the confusion, the authors don't make a conclusion on the results from the lipin 1 knockdown on pg. 13th, and instead only describe the data, making it difficult to follow and understand the logic of the experiment and the interpretation. This is further confounded by the lack of information about the effects on INM DAG and DAG levels in general. Thus, I don't think this experiment is complete, and the authors should either take it out or do the controls that show the levels of DAG and nDAG are impacted under this condition.
8. Finally, it would be helpful for a broader audience to understand physiological or pathological conditions that might mimic oleate + DGATi, which is the most used perturbation, or even CEPT1 KO. For example, it is interesting in Fig. 2 that CCTa will relocate to the INM when cells are loaded with most unsaturated fatty acids, yet PML patches do not form in all of these conditions. The authors state that this suggests that the lack of TAG synthesis (DGATi) is specific to formation of PML patches. However, how this observation ties into conditions where this may be important or the lipid synthesis circuit is unclear.
Minor:
1. Why in Fig. 4 C are PML patches so large? The staining in general looks unique in this panel than the other panels.
2. In Fig. 1 D, there are no scale bars for the magnified cropped images.
3. In Fig. 2, when describing linolenate and linoleate effect how do we know it's because of incorporation into phospholipids that they promote CCTa recruitment? Or is this just assumed?
4. Fig. 7H: mislabeled MG123 should be MG132
5. Fig. 7B and 7I : label CCTa "protein levels" instead of expression to distinguishe from mRNA. Properly label C and D as well to make clear what the read out is.
6. On page 14, the authors write "Approximately 25% of PML CEPT1KO cells" what do they mean by PML CEPT1KO cells? This implies that they are only counting KO cells that are PML +, and goes back to providing a clear explanation of their quantitation.

Dear Dr. Wozniak,

Thank you for providing reviewers comments on our manuscript. We appreciate the time and effort by the reviewers to provide insightful comments and suggestions to improve the clarity and interpretation of experimental results. As suggested, we have removed referencing of 'data no shown' or shown the data in a supplemental figure.

REVIEWER 1

Major comments

Disassembly of PML NBs—The is a good point; we overlooked the quantitation of PML NBs under conditions where PML patches form. We now show in Fig. 1G there is significant 30-40% reduction in nucleoplasmic PML-NBs in DGATi/oleate -treated cells. While this does not support a direct precursor relationship to LAPS or patch formation it does at least show that PML NBs diminish as these alternate structure appear. I agree with the reviewer that the mechanism for this is still unclear and could involve association of PML multimers or monomers with the INM. We have addressed this point in the Discussion (bottom pg.17).

NE rupture or blebbing—The reviewer is correct that the data does not formally show rupture or blebbing without the use of other markers. We have modified the text in the Results and Discussion (Pg. 18) to indicate that PML patches are associated with sites of nuclear lamina depletion after sustained membrane stress rather than rupture (reviewer 2 raised this issue well).

CCTα expression and NE enrichment—I agree with the reviewer that the correlation between CCTα NE enrichment and increased expression is not consistently correlate with PML patch formation. However, it is still potentially important since increased CCTα expression was observed in 3 of the 5 cell lines we surveyed. We failed to clearly explain in the Discussion that cells that assembly nLDs and LAPS (U2OS and Huh7) had PML patches, and increased CCTα NE enrichment and expression whilst results with 3 other cell lines that do not assembly nLDs or LAPS (HeLa, Sh-SY5Y, fibroblasts) were inconsistent. In that context one could argue that CCTα stability at the NE could be linked to nLD and/or LAPS assembly. We have emphasised this point in the Discussion (pg. 18). As suggested, we also condensed Fig. 7 by removing panel E (CCTα expression in response to different 18 carbon fatty acids) and panel D (CCTβ mRNA expression).

Interpretation of futile cycle—Yes, the term futile cycle is a bit overstated without a pulse-chase study of PC turnover. We have removed that term and indicted 'increased phospholipase hydrolysis of PC…' to more accurately describe the response to hyper-activation of CCTα.

Lipin1 knockdown and DAG metabolism-Good point, we have now included that reference to support the observation that Lipin1 depletion did not affect PML patches.

Minor Comments

Fatty acid specificity—Yes, 18:3 was slightly less effective than the other two unsaturated fatty acids but the reason is entirely speculative; could be as simple as reduced uptake of 18:3 or a more complicated mechanism related to its incorporation into DAG and INM phospholipids. We would prefer not to make any more comments on this effect beyond that already noted in the Results section.

PML isoform expression (Figure 9F–G)—We have included molecular mass markers and indicate where the 130 kDa PML isoforms are located. There was a reproducible mobility shift in the PML isoforms in the oleyl-OH treated cells but not with farnesol treatment.

Electrophoretic mobility (Figure 7E). Yes, it should be 'increased' mobility. This sentence is now corrected.

CCTβ analysis (Figure 7C–D)—We have removed the PCYT1B mRNA analysis from Figure 7 since it really serves no purpose in the context of analysis of CCTα expression.

Ubquitination of CCTα—This sentence regarding the absence of ubquitination has been removed and supplemental figure 6 showing that lysosomal degradation is not involved has been added in the Results section (pg.12).

REVIEWER 2

Major points

1. **How do the authors count the percentage of cell with LAPS?--** This was an oversight on our part. A more detailed description of the quantitation of LAPS and PML patches is now included in the Methods section (pg. 21) that was based on a prior publication from our lab (McPhee et.al. 2024).

2. **A better description of how the authors distinguish between PML patches and LAPS in main text would be helpful.--** Basically LAPS in the nucleoplasm have PML plus BODIPY staining (TAG) while the PML patches are at the nuclear periphery, which can be outlined by LMNA/C or Hoechst staining, and are devoid of BODIPY. The two are easy to distinguish based on structure, BODIPY staining and nuclear location. This is more clearly laid out in the Methods section.

3. **Figure 3 is where the paper begins to present a number of smaller points that don't always seem to be critical to the big picture question. One way to tie in the results from figure 3 is to provide the readers with some context on why the different cell lines in particular were chosen?—**The main goal of data in Fig. 3 and SFig. 2 was to compare PML patch formation in cells that do (Huh7, U2OS) or do not (HeLa, SH-SY5Y, Fibroblasts) assemble LAPS and nLDs (see pg. 9). This was then extended to compare the response of these cells in terms of CCTα

expression (Fig. 7 and SFig. 4). It appears that Huh7 and U2OS cells that make nLDs and LAPS have patches, CCTα at the NE and increased expression whist the other three cell lines that lack nLDs and LAPS do not have a consistent response (i.e. HeLa cells have PML patches, NE localization of CCTα but not increased expression). We have more carefully summarized these findings Discussion (pg. 18).

4. **The authors used endogenously tagged Clover - PML to determine the localization of other factors to PML patches in Fig. 5. The lamin A/C clearance is obvious in some cells in top panel of Fig. 5A; however, lamin A/C staining itself looks a bit abnormal and there is no clear rim in the ROI magnification in the bottom panel of Fig5A**. U2OS cell often have a nucleoplasmic vail of LMNA/C that obscures the NE. We have provided new images for Fig. 5A of individual cells that show the depletion of LNMA/C staining at PML patches.

5. Note that although the clearances of laminA/C and somewhat of Emerin are clear, ruptures normally accumulate LaminA/C and Emerin (PMCID: PMC9617480, PMCID: PMC4833568, PMCID: PMC7521799 etc.). This is a good point (also raised by reviewer 1) that we are not showing ruptures but rather sites of lamina depletion/remodelling at PML patches. We have addressed this in the Discussion (pg. 18) as well as the significance statement.

6. **The result in Fig. 6 is confusing because the authors are quantifying LAPS in the Fig. 6A with Oleate treatment yet there are no nLDs in nuclei of the representative cells expressing GFP-PMLI and GFP-PML-IV. Does the expression of these fusion proteins prevent nLDs?—**We did not image nLDs in these experiments because there were no addition laser lines for the lipid dyes. Instead, LAPS were quantified based on the presence of CCTα and Clover-PML on ring structures in the nucleus. We have addressed these issues in legend to Figure 6. The results are consistent with a previous study from our lab (Lee et.al. 2021) that only PML-II was capable of forming LAPS in these PML knockout U2OS cells; PML-I and -IV are ineffective. Similar results were shown by another lab using siRNA silencing of PML expression (Osaki et.al 2016). Because of overexpression of single PML isoforms in a null background, PML NBs and LAPS are more abundant that the wildtype U2OS cells.

7. **Does the depletion of lipin1 affect GFP-DAG localization to the INM?** --Reviewer 1 raised this issue as well and pointed out a publication where in Lipin1 silencing had minimal effect on the NE enrichment of another DAG sensor (Lee et.al. 2023). With this in mind, I would like to keep this data in the manuscript since it does indicate that Lipin1 is not necessary for the patch phenotype (with the noted caveats).

8. Finally, it would be helpful for a broader audience to understand physiological or

pathological conditions that might mimic oleate + DGATi, which is the most used perturbation, or even CEPT1 KO.—Since there is a basal level of PML patches (see Fig. 7) and patches on the INM occur during the production of nLDs and LAPS in Huh7 cells (Soltysik et.al. 2019), they appear to be important in responding to localized stress at the INM. In this report we used treatments and KO cell models to support the concept that it is CES stress that triggers PML association with the INM. We have included this explanation at the end of the Discussion.

Minor points

1. **Why in Fig. 4 C are PML patches so large? The staining in general looks unique in this panel than the other panels.** PML patches can be quite variable in size; there is a large one in Fig. 4 A, and Fig. 2. In addition, some patches are very condensed on the INM while others are more diffuse. We tried to quantify some of this variability but it was too heterogenous and offered no insight into function.

2. In Fig. 1 D, there are no scale bars for the magnified cropped images. We have now included scale bars on the ROI images in that Fig. 1D as well as Fig. 3.

3. In Fig. 2, when describing linolenate and linoleate effect how do we know it's because of incorporation into phospholipids that they promote CCTa recruitment? Or is this just assumed?-This was stated as a potential mechanism to increase CES and the recruitment of CCTa.

4. Fig. 7H: mislabeled MG123 should be MG132—Thanks for picking this out--now corrected.

5. Fig. 7B and 7I : label CCTα "protein levels" instead of expression to distinguish from mRNA. Properly label C and D as well to make clear what the read out is. The y-axis labels have been changed to 'CCTα protein'.

6. On page 14, the authors write "Approximately 25% of PML CEPT1KO cells" what do they mean by PML CEPT1KO cells? This implies that they are only counting KO cells that are PML +, and goes back to providing a clear explanation of their quantitation.—This was a typo. PML is deleted as we are referring to the CEPTKO cells.

We hope that clarifications and changes outlined above satisfy the review's concerns and now make the manuscript suitable for publication in the *MBC*.

Sincerely,

Neale Ridgway, PhD

RE: Manuscript #E25-09-0443R
TITLE: "Membrane curvature elastic stress triggers recruitment of PML-II onto the inner nuclear membrane"

Dear Dr. Ridgway,

Thank you very much for the revised version of your manuscript and for addressing the points raised by the Reviewers. I sent the revised manuscript and your responses to their initial comments back to the Reviewers. They concur with my conclusion that the manuscript is now acceptable for publication. Congratulations to you and your colleagues on this excellent piece of work. Thank you for submitting your paper to MBoC. I hope we see more of your work in the future.

Best regards,

Rick

Richard Wozniak
Monitoring Editor
Molecular Biology of the Cell

Dear Dr. Ridgway:

Congratulations on the acceptance of your manuscript! Thank you for publishing your work in Molecular Biology of the Cell (MBoC).

Within 10 days, an unedited PDF of your manuscript will be published on MBoC in Press, an early-release journal version. The date your manuscript appears on this site is the official publication date.

Your copyedited and typeset manuscript will be scheduled for publication in the next available issue of MBoC. Once your paper is ready for review, our production team will notify you. In the summer of 2024, our production provider will introduce an online proofing solution, which will streamline the process of checking and validating author changes. This will result in improved typesetting quality, enhanced digital content, and a faster overall process.

We invite you to submit images related to your accepted manuscript to be considered for the journal cover.
Cover images must contain color, TIF Format
Size image at 7.4" wide x 5.85" deep
RGB and CMYK color (please submit two separate files, one in each format)
300 dpi resolution

Please also send a 1-paragraph description of the image that a general audience could understand. Include with the caption the name and institution of the individual(s) the image should be attributed to. Please provide this description in a Word file. You can send the file via email or by your favorite file transfer service to mboc@ascb.org.

Authors of Articles and Brief Communications are also encouraged to create a short video abstract to accompany their article when it is published. These video abstracts, known as Science Sketches, are up to 2 minutes long and will be published on YouTube and then embedded in the article abstract. Science Sketch Editors on the MBoC Editorial Board will guide you as you prepare your video. Information about how to prepare and submit a video abstract is available at www.molbiolcell.org/science-sketches. Please contact mboc@ascb.org if you are interested in creating a Science Sketch.

We look forward to publishing your paper in MBoC.
Sign up for MBoC's new content alerts here https://tinyurl.com/3bcsyk3e

Sincerely,

Eric Baker
Journal Production Manager
MBoC Editorial Office
mbc@ascb.org

Reviewer #1 (Remarks to the Author):

The authors have revised the manuscript in a satisfactory manner.

Reviewer #2 (Remarks to the Author):

The authors have addressed my concerns and the manuscript is improved and acceptable for publication.
